# Gram-Positive Bacterial Membrane-Based Biosensor for Multimodal Investigation of Membrane–Antibiotic Interactions

**DOI:** 10.3390/bios14010045

**Published:** 2024-01-15

**Authors:** Samavi Farnush Bint-E-Naser, Zeinab Jushkun Mohamed, Zhongmou Chao, Karan Bali, Róisín M. Owens, Susan Daniel

**Affiliations:** 1 Robert F. Smith School of Chemical and Biomolecular Engineering, Cornell University, Ithaca, NY 14853, USA; sb2535@cornell.edu (S.F.B.-E.-N.); zc83@cornell.edu (Z.C.); 2 Meinig School of Biomedical Engineering, Cornell University, Ithaca, NY 14853, USA; zjm24@cornell.edu; 3 Department of Chemical Engineering and Biotechnology, University of Cambridge, Cambridge CB3 0AS, UK; karankbali@gmail.com (K.B.); rmo37@cam.ac.uk (R.M.O.)

**Keywords:** membrane vesicles, Gram-positive bacteria, daptomycin, supported lipid bilayer, antibiotic sensing, microelectrode array, organic electronic, permeability

## Abstract

As membrane-mediated antibiotic resistance continues to evolve in Gram-positive bacteria, the development of new approaches to elucidate the membrane properties involved in antibiotic resistance has become critical. Membrane vesicles (MVs) secreted by the cytoplasmic membrane of Gram-positive bacteria contain native components, preserving lipid and protein diversity, nucleic acids, and sometimes virulence factors. Thus, MV-derived membrane platforms present a great model for Gram-positive bacterial membranes. In this work, we report the development of a planar bacterial cytoplasmic membrane-based biosensor using MVs isolated from the *Bacillus subtilis* WT strain that can be coated on multiple surface types such as glass, quartz crystals, and polymeric electrodes, fostering the multimodal assessment of drug–membrane interactions. Retention of native membrane components such as lipoteichoic acids, lipids, and proteins is verified. This biosensor replicates known interaction patterns of the antimicrobial compound, daptomycin, with the Gram-positive bacterial membrane, establishing the applicability of this platform for carrying out biophysical characterization of the interactions of membrane-acting antibiotic compounds with the bacterial cytoplasmic membrane. We report changes in membrane viscoelasticity and permeability that correspond to partial membrane disruption when calcium ions are present with daptomycin but not when these ions are absent. This biomembrane-based biosensing platform enables an assessment of membrane biophysical characteristics during exposure to antibiotic drug candidates to aid in identifying compounds that target membrane disruption as a mechanism of action.

## 1. Introduction

The bacterial cell wall is a complex structure that protects bacteria from a hostile environment. This protection extends to limiting membrane permeability against antibiotics and contributing to the rapid development of antibiotic resistance in bacteria. In Gram-positive bacteria, the presence of membrane-active compounds can induce modifications in membrane components and composition that can confer resistance [1]. The Gram-positive bacterial membrane consists of a cytoplasmic membrane composed of phosphatidylglycerol (PG), phosphatidylethanolamine (PE), and cardiolipin (CL), surrounded by a thick peptidoglycan layer that provides a penetration barrier [2]. Alterations in membrane composition such as increased PE concentration have been shown to reduce the effect of cationic antimicrobial peptides due to a reduction in anionic surface charge [3]. In addition, anchored to the membranes of Gram-positive bacteria are lipoteichoic acids (LTAs) which are anionic polymers connecting the peptidoglycan layer to the membrane. D-alanylation [4] and glycosylation [5] of LTAs can also trigger bacterial surface charge modifications, hence impacting the binding of cationic antimicrobial peptides (CAMPs) [5,6]. These membrane modifications, along with changes in the peptidoglycan layer, have resulted in the emergence of Gram-positive bacteria resistant to last-resort antibiotics such as vancomycin [7].

As membrane-mediated antibiotic resistance continues to evolve in Gram-positive bacteria, the development of new approaches to elucidate the membrane properties involved in antibiotic resistance has become critical. One such approach is the use of membrane models as supported lipid bilayers (SLBs), in which a planar lipid bilayer is formed on a solid support such as glass [8,9] or mica [10]. The advantages of using SLB models are numerous, as the two-dimensional planar geometry is compatible with fluorescence microscopy and surface-sensitive techniques such as quartz crystal microbalance with dissipation (QCM-D) [11] and electrochemical impedance spectroscopy (EIS) [12] that can measure membrane biophysical properties [8,9], as well as changes in the membrane due to binding [13,14] or disruption [9,15,16]. SLBs have simplified the challenges associated with isolating surface events *in vivo* to study the impact of membrane composition [17] and antibiotic–membrane interactions [18]. However, typically used reconstituted SLB-based Gram-positive membrane models do not capture native membrane characteristics as these models lack molecular and structural diversity, missing vital components such as teichoic acids and proteins that mediate membrane functionalities.

Recently, we reported the use of membrane-derived vesicles from the outer membrane of Gram-negative bacteria in forming molecularly complex SLBs with the retention of native membrane components such as lipopolysaccharides and outer membrane proteins [9]. Recapitulation of innate membrane behavior in the presence of antibiotics such as polymyxin B has been observed when using SLBs incorporating membrane vesicles (MVs). In this current study, we build a Gram-positive bacterial membrane model using MVs from *B. subtilis* that captures the molecular complexity of this bacteria class. We show that the model membrane retains key biological components such as LTAs, membrane proteins, and bacterial phospholipids. To validate this Gram-positive bacterial membrane model system, its interactions with a membrane-targeting antibiotic, daptomycin, are investigated using two separate analytical techniques, EIS and QCM-D. The interaction of daptomycin with negatively charged phospholipids, such as bacterial phosphatidylglycerol (PG) [19], is specifically mediated through calcium ions (Ca^2+^) [20,21] and corresponds to reduced antimicrobial activity when these ions are absent. Our biosensor captures these calcium-dependent membrane interactions and shows that when calcium is present, membrane permeability is enhanced and two key biophysical properties of the membrane, viscosity and shear modulus, are reduced. These results align with the known mechanism of action for daptomycin and validate that our biomembrane model recapitulates the biological system’s responses to known antibiotics. As such, this biosensor enables a quantitative assessment of antibiotic interactions with the Gram-positive microbial membrane. Given that the membrane is a vital barrier in all bacteria that a drug compound must breech, platforms that focus on the quantification of membrane disruption hold great promise as tools for identifying new classes of antibiotic drugs.

## 2. Materials and Methods

Bacterial strain and vesicle isolation. *Bacillus subtilis* (WT 168) isolates were procured from ATCC (23857). Vesicle isolation protocols were adapted from established protocols [22,23]. Specifically, glycerol stock of *B. subtilis* was used to inoculate 200 mL of brain heart infusion (BHI, Sigma-Aldrich, St. Louis, MO, USA) and grown for 16 h at 37 °C. Bacteria were removed by centrifugation at 4000× *g* for 30 min in a Sorvall ST 8R centrifuge (Thermo Fisher, Waltham, MA, USA). The supernatant was collected and filtered through 0.8 μm, 0.45 μm, and 0.2 μm polyethersulfone (PES) membrane filters (Neta Scientific, Hainesport, NJ, USA) to remove any cellular debris. The filtered supernatant was centrifuged in a Beckman Coulter Ultracentrifuge with a SW28Ti rotor at 28,000 rpm for 3 h. Vesicle pellets were resuspended in phosphate-buffered saline (PBS; 5 mM NaH_2_PO_4_, 5 mM Na_2_HPO_4_, 150 mM NaCl, pH = 7.4) containing 0.2 mM Mg^2+^ and centrifuged for 30 min at 16,000× *g* to remove any remaining impurities. Amicon 100 kDa MW cutoff filter (Neta Scientific) was used to concentrate the vesicles before storing at −80 °C. All centrifugation steps were carried out at 4 °C.

Transmission electron microscopy (TEM). The presence of vesicles in the samples was confirmed using TEM imaging. Carbon-coated copper grids were plasma cleaned for 1 min before adding 5 μL of freshly isolated vesicles. Then, 1.5% uranyl acetate was used for negative staining for visualization using a FEI Tecnai-12 Spirit TEM at 120 kV at the Cornell Center for Materials Research (Cornell University).

Vesicle concentration, size, and surface charge. Nanoparticle tracking (NTA, Malvern Nanosight, Malvern, UK) was used to determine the concentration of the vesicles to ensure consistency across experiments. The size and charge of the vesicles in HEPES were measured using dynamic light scattering (Malvern Zetasizer). Vesicle size distribution was also characterized and confirmed using NTA and TEM.

Total protein content. To quantify the protein concentration of MVs, commercially available Modified Lowry protein assay kit (Thermo Fisher) was used according to the manufacturer’s protocol.

Liposome preparation. Liposomes for inducing the rupture of *B. subtilis* vesicles on planar surfaces were prepared following published protocols. 1-oleoyl-2-palmitoyl-sn-glycero-3-phosphocholine (POPC, Avanti Lipids, Alabaster, AL, USA) was mixed with 1,2-dipalmitoylsn-glycero-3-phosphoethanolamine-*N*-[methoxy(polyethylene glycol)-5000] (DSPE-PEG5000, Avanti Lipids) in a molar ratio of 99.5% POPC and 0.5% DSPE-PEG5000 (referred to as POPC–PEG hereafter) in chloroform as required. Lipids were dried under a stream of nitrogen followed by the evaporation of chloroform under vacuum for 4 h. Liposomes were formed by resuspending the dried lipid films in HEPES buffer (10 mM HEPES (4-(2-hydroxyethyl)-1-piperazineethanesulfonic acid), 150 mM NaCl, pH = 7.4) or Tris buffer (10 mM Tris-HCl, 150 mM NaCl, pH = 7.4) at a final concentration of 2 mg/mL and passing 13–15 times through 50 nm polycarbonate membrane using a mini extruder (Avanti). Liposomes were stored at 4 °C until use.

Bilayer formation and characterization using quartz crystal microbalance with dissipation (QCM-D). A QSense E1 system (Biolin Scientific, Västra Frölunda, Sweden) with a flow chamber and 5 MHz silicon dioxide quartz sensors (QSX 303, Nanoscience Instruments, Phoenix, AZ, USA) was used to perform QCM-D experiments. The sensors were cleaned with 2% sodium dodecyl sulfate (SDS) solution in deionized (DI) water (18.2 MΩ·cm, Siemens PURELAB Ultra (Siemens, Munich, Germany) water purification system) at 40 °C for 1 h and then rinsed with excess amounts of DI water before drying with ultra-high purity nitrogen. Right before use, sensor surfaces were further cleaned in a UV-Ozone Procleaner (Bioforce, Ghent, NY, USA) for 15 min. Data were collected for changes in frequency and dissipation for the first (5 MHz), third (15 MHz), fifth (25 MHz), seventh (35 MHz), ninth (45 MHz), eleventh (55 MHz), and thirteenth (65 MHz) overtones under flow conditions. A peristaltic pump (Ismatec Reglo Digital M2–2/12, QSense, Västra Frölunda, Sweden) was used to flow samples at a rate of 200 μL/min. The chamber temperature was set at 25 °C for all QCM-D experiments.

Before bilayer formation, sensors were equilibrated with HEPES buffer for 5–15 min until a stable baseline was established with the initial changes in frequency and dissipation at 0. MVs were then adsorbed onto the sensor surface until the change in frequency reached −60 Hz before buffer was flowed in to rinse excess vesicles. Next, POPC–PEG at 0.5 mg/mL was pumped into the system to rupture adsorbed MVs for ~1 h under flow conditions. When the change in frequency and dissipation stabilized indicating the completion of rupture, excess liposomes were washed with buffer. For control experiments, POPC–PEG liposomes at a concentration of 0.5 mg/mL were flowed in at 200 μL/min onto the sensor surface for at least 30 min before being rinsed with buffer. As these liposomes are fusogenic, they spontaneously rupture to form a bilayer via vesicle fusion on the sensor surface.

Gram-positive bilayer formation on glass for optical characterization. Glass microscope coverslips (25 × 25 mm, VWR, Radnor, PA, USA) were cleaned by submerging in a solution containing 70% sulfuric acid (BDH, Mumbai, India) and 30% hydrogen peroxide (Sigma-Aldrich) for 10 min and then rinsed with DI water for 30 min. Cleaned coverslips were used directly to characterize supported lipid bilayers (SLBs) by fluorescence microscopy. Polydimethylsiloxane (PDMS, Robert McKeown Company, Branchburg, NJ, USA) wells were prepared by mixing the elastomer with the crosslinker Sylgard 184 (Robert McKeown Company) in a 10:1 ratio and attached to the cleaned glass coverslips right before experiments.

Solution containing ~10^9^ *B. subtilis* vesicles in HEPES was added to PDMS wells attached to the cleaned glass. Following adsorption for 10–12 min, excess and unattached vesicles were washed away with buffer. To rupture the adsorbed MVs and form a planar lipid bilayer, 50 µL of POPC–PEG liposomes were added to the wells at a final concentration of 0.5 mg/mL. Samples were incubated for 1 h to ensure complete rupture and washed with buffer to remove excess liposomes.

For control experiments, POPC–PEG liposomes were added to piranha-washed glass coverslips or plasma-cleaned PEDOT:PSS slides at a final concentration of 0.5 mg/mL. These bilayers were formed via vesicle fusion without the addition of any triggering material. Lipids were incubated for 1 h before the wells were washed with buffer to remove excess material.

Verification of native membrane components on Gram-positive bilayers. The presence of native membrane components on the Gram-positive bilayers was assessed using total internal reflection fluorescence microscopy (TIRFM). The bilayers were specifically tested for the presence of proteins and lipoteichoic acids (LTA). After formation on glass coverslips, SLBs were blocked with 2% bovine serum albumin (BSA, Sigma, St. Louis, MO, USA) for 1 h to reduce non-specific binding. Bilayers were rinsed with buffer before adding specific component-targeting compounds. Alexa Fluor 594 conjugated succinimidyl ester (NHS ester, Invitrogen, Waltham, MA, USA) binds to primary amines and can detect both the presence of proteins with primary amines and phosphatidylethanolamine (PE) in the SLBs. Samples were incubated with 1 µg/mL of the amine-binding fluorophore for 1 h. Before imaging, the bilayers were rinsed thoroughly with buffer to remove excess dye.

Antibody binding experiments were conducted to confirm the presence of LTA (Invitrogen) on the Gram-positive bilayers. Briefly, bilayers blocked with BSA were incubated with 20 µg/mL primary antibody specific to gram-positive bacterial LTA for 1 h and rinsed with PBS. SLBs were then incubated with fluorescently labeled secondary antibody (Abcam, Cambridge, UK) for 45 min and washed with buffer before imaging. As negative controls, POPC–PEG bilayers were also tested for the presence of proteins and LTA in the same ways.

Images were taken with an inverted Zeiss Axio Observer.Z1 microscope with an α Plan-Apochromat 100× objective using 488 nm and 561 nm wavelengths from solid-state lasers. The angle for imaging was 72° for all samples and was controlled by a Laser TIRF 3 Slider (Carl Zeiss, Inc., Oberkochen, Germany). Images were analyzed using ImageJ 1.53a for mean fluorescence intensity (primary amine binding) or particle analysis (antibody binding) using 8–10 images per sample.

PEDOT:PSS microelectrode fabrication. Gold contact pads were patterned on silica wafers using a standard photolithography procedure: exposure, development, deposition, and liftoff. SiO_2_ (200 nm) layer was then deposited on Au patterned wafers using plasma enhanced chemical vapor deposition (PECVD) as an insulating layer. To define the location and dimension of the Au contact pads for the PEDOT:PSS electrodes, a second layer of photolithography was applied, followed by the reactive ion etching of SiO_2_ until the Au surface was exposed. PEDOT:PSS (Clevios PH1000, Heraeus, Hanau, Germany) mixed with 1 *v*/*v* % of (3-glycidyloxypropyl)trimethyloxy-silane (GOPS, Sigma-Aldrich) was then spin-coated at 4000 rpm ubiquitously on wafers, followed by annealing at 140 °C for 30 min to drive off all water. A third layer of photolithography was applied to remove the PEDOT:PSS spun on SiO_2_, leaving PEDOT:PSS only on top of the exposed Au contact pads, taking advantage of the germanium (Ge) hard mask following previously reported protocol [24]. The Ge hard mask (100 nm) on the PEDOT:PSS electrodes was finally removed by immersion in deionized water for 48 h. The dimensions of the electrodes fabricated vary from 120 μm diameter to 420 μm diameter and SLB resistance values reported in this work were normalized with the electrode area.

SLB formation and antibiotic interaction with Gram-positive SLBs on MEA. Fabricated MEAs were soaked in 100 μM KCl solution for at least 2 h, washed with ethanol, and then dried under a stream of N_2_ before light oxygen plasma treatment for ~30 s at low power (6.8 W) under ~200-micron pressure (PDC-32G, Harrick Plasma, Ithaca, NY, USA) to induce hydrophilicity without rendering the surface charge to be too negative. Solution containing ~10^9^ vesicles/mL was diluted with 1 mg/mL POPC liposomes (without DSPE-PEG5000) in Tris buffer (10 mM Tris, 150 mM KCl, pH = 7.4) at a ratio of 1:20 and the mixture was sonicated in a bath sonicator (B2500A-DTH, VWR) for 20 min to induce fusion [25,26]. The MV–liposome mixture was introduced to the freshly plasma-treated MEA and incubated for 1 h to allow for the self-assembly of the Gram-positive SLB. After 1 h, excess MV–liposome mixture was washed away with the HEPES before recording the electrical signal. For studying the impact of antibiotic addition, daptomycin in HEPES at the desired concentrations was added to both the Gram-positive SLBs and the POPC SLBs and incubated for 1 h before taking EIS measurements. HEPES was supplemented with 3 mM Ca^2+^ as required. SLB formation and subsequent interactions with daptomycin were tested at room temperature.

Electrochemical impedance spectroscopy (EIS) measurement setup. EIS was performed using a potentiostat (Autolab PG-STAT204, Utrecht, The Netherlands) in a three-electrode configuration with Ag/AgCl and Pt mesh being used as the reference and counter electrodes, respectively. Each PEDOT:PSS electrode in a single array was sequentially used as the working electrode. An AC voltage of 50 mV and a DC voltage of 0 mV versus OCP were applied. The responding current was recorded within the frequency range 1–10^6^ Hz, with 10 data points per decade (equally spaced on a logarithmic scale). Depending on the experiment, HEPES (10 mM HEPES, 150 mM NaCl, pH = 7.4) buffer with or without 3 mM Ca^2+^ was used as the electrolyte. EIS measurements were performed for electrode baseline (no SLB), after SLB formation according to the above-mentioned protocol, and following 1 h incubation with the desired concentration of daptomycin in HEPES with or without 3 mM Ca^2+^. Data were collected and analyzed using NOVA 2.1.3 software (Metrohm Autolab, Utrecht, The Netherlands).

Antibiotic interaction with Gram-positive SLBs using QCM-D. For studying antibiotic interactions using QCM-D, Gram-positive bilayers were formed and maintained at 25 °C. To study membrane interactions with antibiotics, daptomycin (VWR) at 50 μg/mL was pumped into the chamber for 30 min following bilayer formation. Changes in frequency and dissipation were recorded before and after antibiotic addition for the following cases: Gram-positive bilayer with daptomycin in the presence of Ca^2+^, Gram-positive bilayer with daptomycin without Ca^2+^, and POPC–PEG bilayer with daptomycin in the presence of Ca^2+^. HEPES supplemented with 3 mM Ca^2+^ and HEPES without Ca^2+^ were used as the buffers for studying SLB interactions with daptomycin.

Antibiotic interactions with the Gram-positive bilayer were modeled using a Voigt–Voinova two-layer model [27] to determine the thickness, viscosity, and shear modulus of both the top and bottom layers (the details for modeling are provided in Supplementary Materials Section S5). The Voigt one-layer model was used to calculate viscoelastic variables for the POPC–PEG SLB using software provided by QSense. All viscoelastic properties were calculated using the third, fifth, seventh, ninth, eleventh, and thirteenth overtones for both models. Changes in frequency and dissipation are plotted normalized by overtone number.

## 3. Results and Discussion

### 3.1. Naturally Secreted Nanoscale Vesicles from B. subtilis Retain Native Characteristics

**Figure 1:**
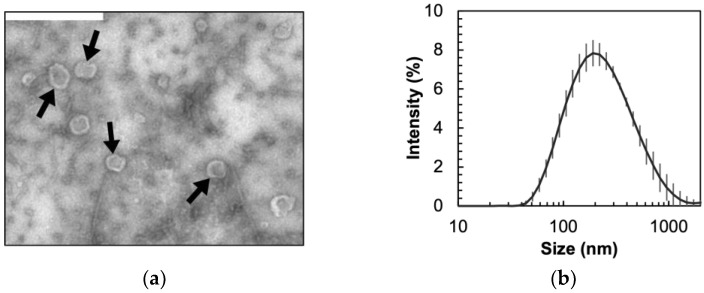
(**a**) TEM image of negatively stained MVs isolated from *B. subtilis* shows intact vesicles indicated by black arrows. Scale bar represents 500 nm. (**b**) Dynamic light scattering (DLS) results for vesicle size distribution. Error bars represent standard deviation.

Even though vesicle production in Gram-positive bacteria is not as well studied as outer membrane vesicles (OMVs) from Gram-negative bacteria, many published works show that MVs are produced by several Gram-positive bacterial species [23,28,29,30,31]. Recent work by Brown et al. [28] demonstrates that MVs are spontaneously produced and released by wild-type (168) and environmental (3610) strains of *B. subtilis,* with comprehensive proteomic analysis showing retention of cellular and membrane proteins. In this work, we isolated MVs from *B. subtilis* WT 168 strain (without induction) and characterized these particles. Size distribution of the vesicles obtained through transmission electron microscopy (TEM), dynamic light scattering (DLS), and nanoparticle tracking analysis (NTA) demonstrate heterogeneity in the MV population and is similar to previously reported distributions for bacterial vesicles [28,29,31,32]. The TEM images in Figure 1a and Figure S1a confirm the morphology of intact vesicles with an average size of 80–230 nm, after applying a 1.27 correction factor for converting 2D size distribution to 3D size distribution of spheres [33]. The vesicle size distribution obtained using light scattering, as shown in Figure 1b and NTA experiments included in Supplementary Figure S1b, estimate the average vesicle sizes to be slightly higher than the value calculated from TEM images. DLS and NTA measure the hydrodynamic diameter of the vesicles in buffer leading to this overestimation, as reported in previous research [28,34,35]. NTA was further used to quantify vesicle concentration after each isolation to ensure a consistent number of vesicles were used throughout different experiments.

Laser Doppler electrophoresis was used to determine the surface charge of vesicles and the ζ potential was found to be −18.77 ± 1.61 mV in HEPES buffer. This is consistent with published values for the whole *B. subtilis* cells at −18.55 mV in similar buffer conditions [36]. We also confirmed the presence of membrane proteins in the isolated vesicles with SDS-PAGE, as shown in Supplementary Figure S1c. To quantify total protein in the vesicles, we used the Modified Lowry assay and found the protein content to be in the range of 5.39–7.58 mg/mL across MVs from different batches of isolation. The zeta potential and protein analysis confirm the retention of native constituents, such as bacterial lipids and proteins, in the isolated MVs.

### 3.2. MVs Can Be Ruptured to Form Gram-Positive SLB

**Figure 2:**
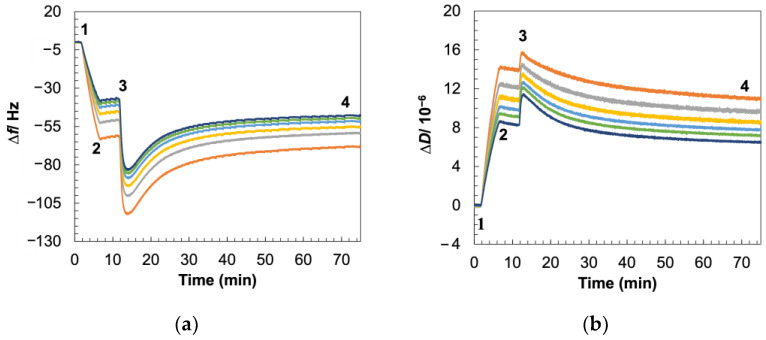

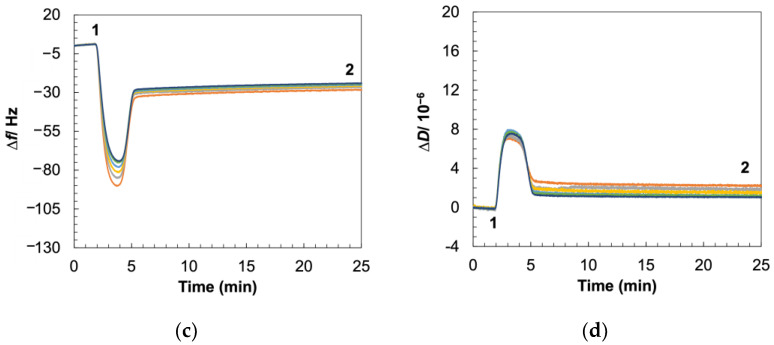
Changes in Δf and ΔD with time during bilayer formation using QCM-D. (**a**,**b**) SLB using *B. subtilis* MVs was formed by (1) flowing in vesicles for adsorption, (2) rinsing excess vesicles with buffer, (3) adding rupture vesicles, and (4) washing away excess material with buffer upon stabilization of signal. (**c**,**d**) The POPC–PEG SLB was formed by (1) flowing in vesicles and (2) washing away excess vesicles with buffer after completion of rupture indicated by signal stabilization. Different colors represent different overtones: orange (3rd = 15 MHz), grey (5th = 25 MHz), yellow (7th = 35 MHz), light blue (9th = 45 MHz), green (11th = 55 MHz), and dark blue (13th = 65 MHz).

SLBs are commonly formed on planar support via vesicle fusion mediated by favorable van der Waals and electrostatic interactions between the support and liposomes [37,38]. However, due to the complexity of the composition and highly negative surface charge, MVs from bacteria do not spontaneously rupture on most surfaces to form planar bilayers [8,9,15]. To overcome this, we have used more fusogenic, zwitterionic reconstituted liposomes to rupture adsorbed MVs through bilayer edge interactions, as established in previous work [8]. In this work, we tested PEGylated POPC (POPC–PEG) as the fusogenic liposome to rupture *B. subtilis* vesicles on a variety of substrates, including quartz crystal surfaces (Figure 2), glass, and PEDOT:PSS. Details about the formation of Gram-positive SLBs on glass and PEDOT:PSS are provided in Supplementary Materials Section S2. Although PC lipids are predominantly found in mammalian membranes, the zwitterionic nature of these liposomes ensures minimal interference with antibiotics [39]. The polyethylene glycol (PEG5k, 0.5 mol%) increases the water gap between the bilayer and the substrate and reduces the possibility of denaturing transmembrane proteins by acting as an inert cushion [8]. PEGylated lipids in the concentration range used in this work are in the “mushroom” regime, having a globular shape with little impact on bilayer mobility and diffusion [40,41].

We first validated the formation of Gram-positive SLBs using the isolated MVs from *B. subtilis* via quartz crystal microbalance with dissipation (QCM-D, Figure 2) and fluorescence recovery after photobleaching (FRAP, Supplementary Materials Section S2). QCM-D is a widely used tool for quantitively monitoring the formation of supported lipid bilayers and their interactions with membrane-acting compounds by recording changes in resonance frequency (Δ*f*) and dissipation (Δ*D*) of a piezoelectrically excited silicon dioxide sensor [42,43,44,45]. These changes are logged at multiples of the fundamental harmonic of the sensor (overtones) which correspond to different penetration depths of the signal, with lower overtones having higher penetration depths. While Δ*f* corresponds to changes in mass adsorbed on the surface, Δ*D* corresponds to film stiffness measured in terms of the ability of the material on the sensor to dissipate acoustic energy [46,47].

As vesicles flowed into the QCM chamber, we recorded a shift in Δ*f* indicating an increase in mass adsorbed on the sensor surface, while the change in ΔD implied that the added material is viscoelastic (Figure 2a,b). To maximize the MV content in the final SLB while still maintaining enough surface area for rupture to occur, we allowed MVs to adhere until Δ*f* reached ~60 Hz, as it has been previously demonstrated that vesicle crowding on the sensor surface prevents rupture and self-assembly of the native membrane vesicles into the planar bilayer structure [9]. Next, POPC–PEG was added to initiate SLB formation via vesicle fusion. Initially, we recorded further changes in Δ*f* and Δ*D* corresponding to this introduction of additional viscoelastic material to the sensor surface. The addition of POPC–PEG induces the rupture of the MVs through bilayer edge interaction and the consequent release of the coupled water mass originally confined inside the intact vesicles resulted in an upsurge in Δ*f* and a descent in Δ*D*, a telltale sign of supported lipid bilayer formation [48,49]. To ensure that the MVs indeed rupture to form SLBs when POPC–PEG is introduced to the system, we utilized fluorescence microscopy to visualize the process (Supplementary Materials Section S2 and Video S1). Furthermore, the theoretical MV rupture percentage on the QCM-D sensor surface was evaluated to be ~78% which is detailed in Supplementary Materials Section S3. The completion of the rupture process is indicated by the stabilization in Δ*f* and Δ*D* values as MVs and POPC–PEG liposomes fuse to form SLBs on the sensor surface.

The clear separation of Δ*f* and Δ*D* values over different overtones, along with the much higher Δ*D* value for the bacterial SLB in contrast to the POPC–PEG SLB (Figure 2c,d), can be attributed to the former formulation resulting in a more viscoelastic and less rigidly packed film than the latter case. Additionally, compared to the POPC–PEG SLB, the final Δ*f* values for the Gram-positive SLBs are much lower, pointing to the successful incorporation of heavier native membrane materials such as proteins and polymeric sugar chains into the lipid bilayer. Indeed, the mass of the POPC–PEG SLB was estimated using the one-layer Voigt viscoelastic model to be 744.6 ± 101.4 ng/cm^2^, while the mass Gram-positive SLB was estimated using the two-layer model to be 1567.3 ± 554.9 ng/cm^2^. As discussed later in this manuscript, the one-layer model fails to capture the viscoelastic behavior of the latter bilayer. The presence of LTA in the latter SLB (confirmed in the next section) also validates the application of the two-layer model to characterize this bilayer. The greater mass of the Gram-positive SLBs, again, indicates the integration of heavier bacterial membrane components into this SLB.

### 3.3. Gram-Positive Bilayers Retain Bacterial Membrane Components

The incorporation of native membrane components into SLBs has been demonstrated previously for multiple Gram-negative bacterial species [8,9,15]. To prove that our Gram-positive lipid bilayer preserves molecular heterogeneity of the native membrane that regulates lipid packing, stabilization, and membrane function, as implied by our QCM-D results discussed above, additional investigation is needed.

**Figure 3:**
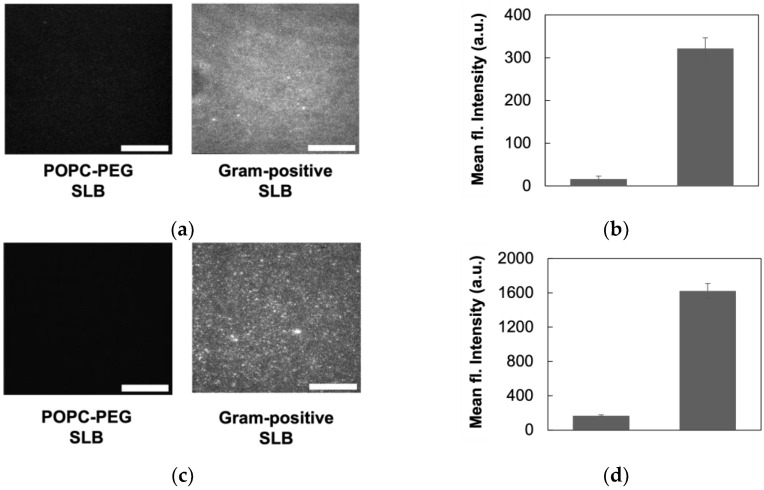
Membrane protein and LTA retention in Gram-positive SLBs. (**a**) TIRFM images for Alexa Fluor 594 succinimidyl ester binding to POPC–PEG and Gram-positive bilayers. Higher fluorescence is indicative of the presence of primary amines found in proteins. (**b**) Mean fluorescence intensity from TIRFM images for primary amine binding. (**c**) TIRFM images for anti-LTA antibody binding to POPC–PEG and Gram-positive bilayers. (**d**) Mean fluorescence intensity from TIRFM images for antibody binding. For quantification, images (*n ≥* 8) obtained using TIRFM were analyzed via ImageJ. Error bars represent standard deviation. Scale bars represent 20 μm.

Since the retention of proteins in Gram-positive MVs has been verified in current work using Modified Lowry assay (Section 3.1) and SDS-PAGE gel (Supplementary Figure S1c), as well as in previous research [23,28], we used Alexa Fluor 594 conjugated succinimidyl (NHS) ester (Life Technologies, Carlsbad, CA, USA) to test for the presence of bacterial proteins in our model system. Fluorescently labeled NHS esters can covalently couple to free primary amines and are used to label proteins as they are prevalent sources of free primary amines on the outer leaflet of the bacterial membrane [50,51]. Upon exposing our SLBs to succinimidyl esters and visualizing with TIRFM, we observed significantly higher staining in the Gram-positive bilayers compared to POPC–PEG bilayers (Figure 3a,b). This indicates the presence of amine residues only in MV-derived bilayers, but not in the lipid-only bilayer controls. Phosphatidylethanolamines (PE) can also contribute to the fluorescent signal as they can also couple to the NHS ester. However, the relatively low PE content of *B. subtilis* membrane [52,53,54] points to some of the recorded fluorescent signals being attributed to the presence of proteins. Regardless, the source of both the PE lipids and proteins originates from the bacterial membrane demonstrating the retention of native components in the Gram-positive SLB.

To determine the presence of lipoteichoic acids (LTA) in our Gram-positive bilayers, we used an anti-Gram-positive bacteria antibody (Life Technologies) that binds specifically to bacterial LTA, which are anionic cell surface polymers anchored to the cell membrane [2]. For visualization of antibody binding to LTA using TIRFM, we used a fluorescently labeled secondary antibody that binds to the primary anti-LTA antibody. We observed a significantly higher fluorescent signal from our Gram-positive bilayer compared to the POPC–PEG bilayer (Figure 3c,d). More TIRFM images from the LTA binding experiment are included in Supplementary Figures S5 and S6. Our results demonstrate that the bilayers formed using *B. subtilis* MVs retain the LTA material of the bacterial membrane. Additionally, the access of the antibody to LTA suggests that these molecules mostly maintain native orientation in the Gram-positive SLBs, with the polymer chain facing the bulk phase.

### 3.4. Viscoelastic Characterization of Gram-Positive Bilayer

**Figure 4:**
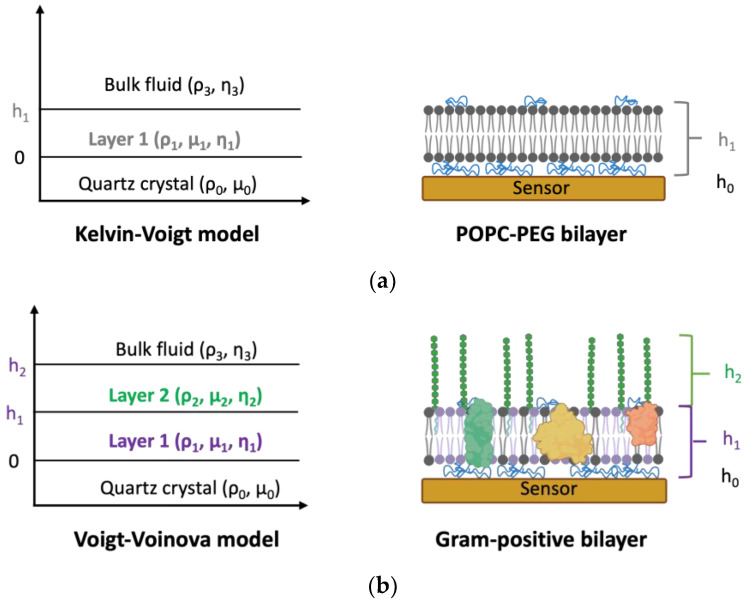
Graphical representations of the (**a**) one-layer Kelvin–Voigt model for the POPC–PEG bilayer (grey) and (**b**) two-layer Voigt–Voinova model for the Gram-positive bilayer. Note that the LTA (green) is not drawn to scale with the bilayer (purple) thickness.

QCM-D data obtained during Gram-positive SLB formation were used to determine membrane viscoelastic properties such as thickness, shear modulus, and viscosity, providing important insights into the impact of antibiotic interactions on membrane properties. Due to the highly viscoelastic nature of bilayers (dissipation factor, ΔD >> 10^−6^), we applied viscoelastic models to accurately estimate SLB properties. For modeling the POPC–PEG bilayer, the one-layer Voigt model [27] built into the Qtools software 4.4 was used. This model considers the viscoelastic film on the sensor surface as a homogeneous layer and provides information about the mechanical properties of the entire layer (Figure 4a). However, the Gram-positive bilayer is inherently heterogeneous as native bacterial membrane components, including LTA molecules which extend as a second “layer” above the lipid bilayer. Therefore, to appropriately model this system, we used the two-layer Voigt–Voinova model [27], which is used to evaluate viscoelastic properties such as thickness, viscosity, and shear modulus of heterogeneous films on QCM-D sensors (Figure 4b). This allowed us to evaluate structural changes occurring at different levels across the depth of the bilayers in detail, as discussed below. Detailed description of QCM-D data modeling is provided in the Supplementary Information (Section S5), along with the fitting of the experimental data to different models (Figure S7). The estimated values for bilayer properties obtained using the corresponding models are listed in Table 1.

Upon comparing the magnitude of the viscoelastic properties of the different layers of the Gram-positive bilayer with the POPC–PEG bilayer, we surmise that layer 1 in our model corresponds to the lipid bilayer, while layer 2 corresponds to the polysaccharide chains of the LTA molecules. The estimated thickness of the POPC–PEG bilayer is slightly higher than the phospholipid bilayer thickness (4 nm) reported in the literature [55]. The presence of PEG chains, with a globular diameter of ~5 nm [56] at the concentration used for this study, adds to the thickness of the layer resulting in this increase, but because of the dilute concentration, it leads to an apparent thickness of approximately 7 nm. The simulated thickness of the Gram-positive bilayer matches the lipid-only SLB, indicating that this layer extends only up to the top of the outer leaflet of the bilayer segment, as demonstrated in Figure 4b. The calculated viscosity of the Gram-positive layer 1 is lower than the reported values of *B. subtilis* membrane viscosity (~1000 cP) [57], which is expected due to the absence of the peptidoglycan layer, which provides structural support in intact bacterial cells. However, the SLB viscosity values for both bilayers are in the same order of magnitude as published values for lipid bilayers [58,59]. The calculated shear modulus values of the bilayers (290–360 kPa) were found to be in the range typical for phospholipid bilayers [8,9,60]. The viscosity and shear modulus of the Gram-positive layer 1 are both slightly higher than the bilayer comprising only lipids, indicating lower fluidity of the former, potentially due to the presence of native membrane material.

The second layer of the Gram-positive bilayer likely represents the polyglycerol phosphate chain of the type I LTA typically found in *B. subtilis* [61]. The modeled thickness and viscosity of this layer are within the range of published values of ~8–17 nm [62] and ~1 cP [63] for LTA, respectively. The viscosity and shear modulus of this layer are both lower compared to layer 1, presumably due to the low density and less-ordered alignment of the polymeric chains in the SLB platform in contrast to the densely packed bottom layer.

### 3.5. Probing Membrane-Targeting Antibiotic Activity Using Electrochemical Impedance Spectroscopy (EIS)

Daptomycin is an anionic, amphiphilic lipopeptide that targets Gram-positive bacterial cell membranes [20,64]. Calcium ions (Ca^2+^) mediate daptomycin interaction with negatively charged phospholipids [19], such as bacterial phosphatidylglycerol (PG) [20,21], leading to depolarization and destabilization of the membrane [64,65,66]. The strong dependence of daptomycin antibacterial activity on the presence of Ca^2+^ ions [67] provided us with a clear control case to validate our platform as well. Even though daptomycin has been reported to trigger stiffening of PE:PG SLBs by lowering the phase transition temperature of the lipid mixture after PG sequestration by daptomycin [68], using fluorescence microscopy, we detected no phase separation in the Gram-positive SLB at room temperature (Supplementary Figure S8). While these observations are limited by the diffraction limit (200–250 nm), the presence of POPC lipids in our Gram-positive membrane model, along with the lower concentration of PE lipids in the *B. subtilis* native membrane [52,53,54], may also have prevented widespread lipid demixing during our experiments. As discussed next, we observed an overall destabilization of the membrane upon daptomycin addition to the Gram-positive bilayer, coincident with the established mechanism of action of this antibiotic [64,65,66].

**Figure 5:**
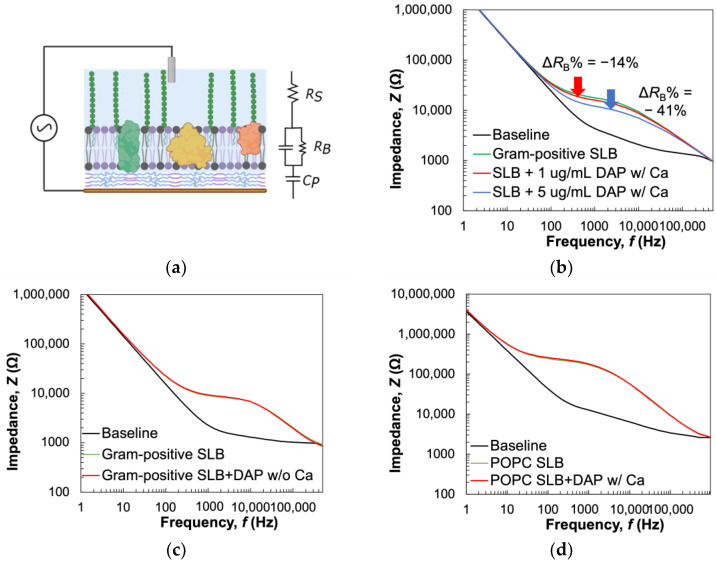
EIS monitoring of SLB interactions with daptomycin. (**a**) Schematic representation of SLBs formed on PEDOT:PSS electrodes, with an equivalent circuit used for fitting impedance data. (**b**–**d**) Representative Bode plots showing impedance response**s** of SLBs upon addition of daptomycin (DAP) for (**b**) Gram-positive SLB in the presence of Ca^2+^, (**c**) Gram-positive SLB in the absence of Ca^2+^, and (**d**) POPC SLB in the presence of Ca^2+^.

We used electrochemical impedance spectroscopy (EIS) to directly assess the permeability change of the Gram-positive SLBs in the presence of daptomycin due to the high sensitivity of the electrical measurement technique compared to other analytical methods such as fluorescence microscopy. To evaluate the interactions of our model system with daptomycin, we formed the Gram-positive bilayers on microelectrode arrays (MEAs). In recent years, the application of MEAs has seen an exponential rise in the field of electrical monitoring and stimulation of biological material including live cells [69,70]. Significant advances in MEA fabrication techniques have driven researchers to pursue the development and optimization of strategies for modifying electrode surfaces to promote or inhibit biomaterial attachment based on specific applications [69,70,71]. While different approaches, such as covalent coupling using synthesized [72] or DNA tethers [73], crosslinking [74], self-assembly [75], etc., have been employed to functionalize surfaces for adhesion enhancement, hydrophobic organosilane molecules are used as passivating layers for controlling biomolecule deposition [69]. In this work, we used an organic, conjugated polymer support, PEDOT:PSS, to facilitate SLB formation and capture the electrical properties of SLBs using EIS scans, as depicted in Figure 5a. PEDOT:PSS is a conducting polymer (CP) widely used as the electrode material in bioelectronics due to its biocompatibility [12] and low impedance [14] to increase the signal-to-noise ratio. Lipid bilayers are usually modeled as a capacitor and resistor connected in parallel, making the EIS signal a signature “chair shape” when SLBs are formed on top of a PEDOT:PSS electrode, as shown in Figure 5b–d. EIS measures impedance, which reflects how easily charged ions can travel to or from the electrode in response to the alternating voltage applied. Hence, ion permeation regulated by SLBs formed on PEDOT:PSS electrodes can be assessed directly via the changes in SLB impedance. In addition, the PEDOT:PSS electrode can swell and provide cushioning to the SLB components to support their mobility and functionalities [76], and its transparency also allows for optical characterizations [12] of SLBs independent of the electrical monitoring.

In this work, PEDOT:PSS MEAs were fabricated following the procedure described in the experimental section. POPC lipids were used to facilitate MV rupture and the formation of Gram-positive SLBs on the microelectrodes. The formulation of PEDOT:PSS used for the microelectrode fabrication results in a film of ~100–200 nm [77] providing adequate cushioning for the SLB components, eliminating the need to include PEG5k lipids. The native vesicles were fused with reconstituted liposomes using sonication [25,26] for SLB formation on the conditioned PEDOT:PSS surface (details are provided in the Materials and Methods section). We first investigated the changes in membrane impedance resulting from daptomycin acting on Gram-positive bilayers in the presence of Ca^2+^. As shown in Figure 5b, prior to SLB formation, the signal exhibits a “hockey stick” baseline signal indicative of the RC in series circuit. Upon the formation of Gram-positive SLB, the signal shifted from the baseline (black) to the chair shape (green), confirming the bilayer formation. The impedance response of the system was then used to extract values for specific membrane electrical properties, namely, resistance by fitting the recorded data to the equivalent circuit model shown in Figure 5a. The electrolyte was modeled as a resistor (R_S_) in series with the working electrode, PEDOT:PSS, which was modeled as a capacitor (C_P_). SLBs were modeled as a resistive element (R_B_) in parallel with a capacitive element (C_B_) [15,78]. We calculated the average R_B_ and C_B_ values for the Gram-positive SLB to be 6.8 ± 3.9 Ω × cm^2^ and 1.6 ± 0.7 μF/cm^2^, respectively. Changes in membrane integrity resulting from disruptions caused by daptomycin interactions can be directly evaluated through changes in membrane resistance to ionic flow (ΔR_B_) and, therefore, we used this parameter as a measure of such interactions. Upon daptomycin addition (1 µg/mL) to the Gram-positive bilayer in the presence of Ca^2+^, we recorded a significant drop in membrane resistance (ΔR_B_ = −15.1 ± 7.8%). We believe this is due to the known interactions of daptomycin with PG-containing membranes in the presence of Ca^2+^ [39,79,80], which induces transmembrane ion conduction [81,82,83,84], thereby reducing overall impedance. When a higher concentration of the antibiotic (5 µg/mL) was added, the membrane resistance reduced further to 40.7 ± 10.9%. With the addition of an even higher concentration of daptomycin (10 µg/mL), no significant reduction in membrane resistance was recorded (Supplementary Figure S9). A similar saturation effect of daptomycin activity has been reported previously [68] and is indicative of the nature of disruption caused by daptomycin activity. Unlike antimicrobial peptides (AMPs) that cause rapid destruction of the membrane [85], this lipopeptide antibiotic depolarizes the membrane potential, leading to transient ion leakage without pore formation [80].

As controls, in the absence of Ca^2+^ (Figure 5c) or when daptomycin was added to the POPC bilayer (Figure 5d), insignificant impedance changes indicate no disruption or permeabilization of the SLB occurred in either case. Combining with all three systems we tested, we confirmed interactions of daptomycin were selective to Gram-positive SLB in the presence of Ca^2+^, consistent with our QCM-D data and well-established antibiotic studies. It is worth mentioning that the POPC:MV ratio used for the SLB formation on the MEAs was optimized to maximize native component incorporation (Supplementary Figure S10), resulting in more ion leakage through the Gram-positive SLB (via defects or ion channels). In contrast, the higher resistance (41.8 ± 1.1 Ω × cm^2^) of POPC SLB indicates a denser packing of lipid molecules in this homogeneously composed synthetic SLB. To rule out the prevalence of unruptured MVs leading to lower resistance, we used fluorescence microscopy to confirm MV rupture (Supplementary Materials Section S2). In addition, the higher capacitance of the Gram-positive bilayer compared to the POPC bilayer (1.1 ± 0.4 μF/cm^2^) reflects the presence of an LTA layer extending beyond the phospholipid bilayer section of the bacterial membrane model. Such trends are consistent with previously published resistance of native and synthetic SLBs on PEDOT:PSS using only zwitterionic lipids [15,78,86]. The values of electrical resistance and capacitance for SLBs, along with changes in membrane resistance after interactions with daptomycin under different conditions, are listed in Table S2.

Our electrical results demonstrate that the vesicle-derived bioelectronic platform provides key insights into antibiotic activity, such as the specificity of daptomycin interactions with the cellular membrane requiring the presence of both negatively charged PG lipids and Ca^2+^ and the impact of these interactions on the membrane. In addition, the in-vitro detection of antimicrobial activity of the molecule at concentrations corresponding to the reported minimum inhibitory concentration (MIC) of daptomycin (1 μg/mL) against *B. subtilis* WT 168 strain reported in the literature [87] highlights the sensitivity of the developed bioelectronic sensor in recapitulating native membrane interactions. To validate our electrical results, we next probed the interactions of daptomycin with our model membrane systems using QCM-D.

### 3.6. Viscoelastic Changes Recapitulate Mechanism of Daptomycin Interactions with Gram-Positive Membrane

**Figure 6:**
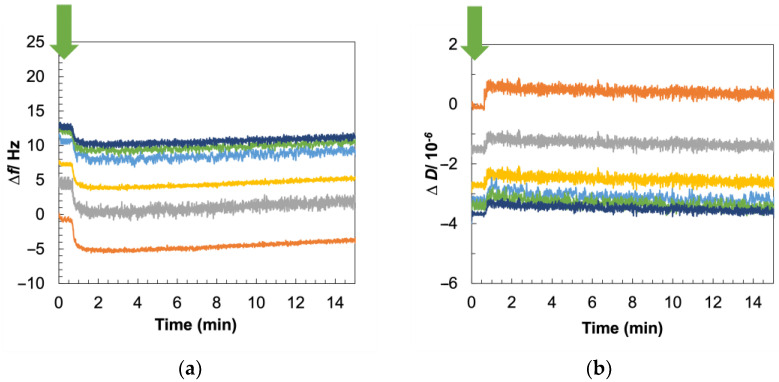
Daptomycin interaction with Gram-positive SLB using QCM-D in the presence of Ca^2+^. The SLB is washed with buffer supplemented with Ca^2+^ before daptomycin addition to isolate the impact of the antibiotic. Representative plots monitor (**a**) Δ*f* and (**b**) Δ*D*, with time for the process. The time of daptomycin addition is marked with green arrows. Upon daptomycin addition, a drop in Δ*f* indicating insertion of the antibiotic into the bilayer and a rise in Δ*D* indicating destabilization of the bilayer, were recorded. Values are normalized post-SLB formation. Different colors represent different overtones: orange (3rd = 15 MHz), grey (5th = 25 MHz), yellow (7th = 35 MHz), light blue (9th = 45 MHz), green (11th = 55 MHz), and dark blue (13th = 65 MHz).

QCM-D provides information about the impact of membrane-acting compounds such as daptomycin on membrane viscoelasticity, and we used this tool to corroborate our results from the electrical experiments. After forming Gram-positive bilayers on QCM-D sensors, as described in Section 3.2, we introduced a flow of daptomycin in HEPES supplemented with Ca^2+^ after washing the bilayer with the same buffer to drive away excess liposomes. As shown in Figure 6a,b, after antibiotic addition, we recorded a decrease in Δ*f*, indicating the accumulation or aggregation of daptomycin with insertion of its hydrophobic tail into the lipid film. We also observed an increase in Δ*D*, which can be attributed to the induction of membrane depolarization and destabilization by daptomycin. Our results showing the loss of SLB rigidity due to daptomycin molecules interacting with the negatively charge phospholipids in SLB are in alignment with previous research [66,81,83,88,89]. Additional analyses of the data included in Supplementary Figure S11 show that the lipopeptide–membrane interaction is a two-step process, with the changes in Δ*f* and Δ*D* being more pronounced at the lower overtones, implying that daptomycin interacts preferentially with the outer leaflet of the bilayer, as the lower overtones correspond to the top of the lipid bilayer, as has been suggested by prior research [67,79,87,90].

When we tested the activity of daptomycin, either in the absence of Ca^2+^ against Gram-positive bilayers or in the presence of Ca^2+^ against POPC–PEG bilayers, we observed no changes in Δ*f* or Δ*D* (Supplementary Figures S12 and S13). For these control experiments, the bilayers were washed with buffer without Ca^2+^ in the former case and buffer supplemented with Ca^2+^ in the latter case, before the antibiotic addition to specifically isolate the impacts of Ca^2+^ and native materials, respectively. These results suggest that the changes in Δ*f* and Δ*D* reported in Figure 6 are specifically due to the presence of both bacterial membrane components in the SLB platform and Ca^2+^ in the membrane environment, validating the capability of our model membrane system in recapitulating Gram-positive bacterial membrane interactions with daptomycin, since daptomycin requires Ca^2+^ for charge neutralization to interact with PG [20,87].

**Figure 7:**
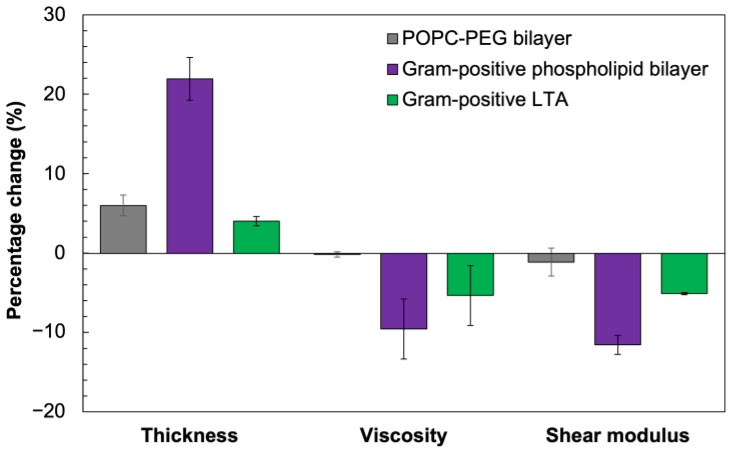
Impact of daptomycin interaction on membrane viscoelastic properties in the presence of calcium ions. Grey bars correspond to the POPC–PEG bilayer, purple bars correspond to the Gram-positive lipid bilayer, and green bars correspond to the polymeric chain component of LTA extending beyond the bilayer. Changes in modeled parameters, e.g., thickness, viscosity, and shear modulus, after antibiotic addition, are presented as percentage changes normalized with respect to the values for each parameter before the antibiotic was added. Error bars represent standard error from 3 independent experiments.

Next, we evaluated the impact of daptomycin addition on the viscoelastic properties of the POPC–PEG bilayer, as well as both layers of the Gram-positive bilayer in the presence of Ca^2+^. We employed the one-layer model for the former and the two-layer model for the latter SLB to fit the recorded Δ*f* and Δ*D* data. The applicability of these models to estimate changes in thickness, viscosity, and shear modulus resulting from interactions with membrane-acting antibiotics has previously been established [8,9]. Details about the modeling and the fit of the experimental frequency and dissipation values are provided in Supplementary Materials Section S5. To account for the batch-to-batch variability in the amount of bacterial material present in the bilayers, we normalized the changes in the viscoelastic parameters of the membrane following the addition of antibiotics for each independent experiment with respect to the parameter values before the changes were triggered (Figure 7).

As illustrated in Figure 7, the viscoelastic properties of layer 1 are highly impacted by the addition of the antibiotic in the presence of Ca^2+^ due to the preferential interaction of the daptomycin–Ca^2+^ complexes with the anionic PG lipids in this layer [64,65,66]. The thickness of layer 1 increased by 21.9 ± 2.7%, indicating that the antibiotic–Ca^2+^ complexes are adsorbed on the membrane, with the lipid tail of daptomycin being inserted into the bilayer. We observed a 9.6 ± 3.8% decrease in the viscosity and an 11.5 ± 1.2% decrease in the shear modulus of layer 1 (phospholipid bilayer). These changes denote the loss of fluidity and acyl chain alignments of the phospholipids in layer 1 after daptomycin interaction with PG, destabilizing the Gram-positive bilayer. For layer 2, the model estimated small changes in the viscoelastic properties—a 4.0 ± 0.6% increase in thickness along with 5.3 ± 3.8% and 5.1 ± 0.2% reductions in viscosity and shear modulus, respectively. These changes were potentially induced by the destabilization of the lipid anchors of LTA molecules embedded into the bilayer from daptomycin activity. In contrast, the addition of daptomycin increased the thickness of the POPC–PEG bilayer by 5.9 ± 1.3%. However, no significant changes in either viscosity or shear modulus were estimated from our model fitting, indicating some non-specific adsorption of the antibiotic into the POPC–PEG bilayer.

Our results from evaluating the impact of daptomycin addition to the Gram-positive SLB demonstrate the ability of this biosensor to recapitulate the Ca-dependent interactions of the membrane-targeting antibiotic with the native Gram-positive membrane. These QCM-D experiments uphold our interpretations of the electrical data, establishing the developed bacterial membrane model as a useful tool for investigating the impact of native bacterial membrane interactions with membrane-acting compounds without the need for living microorganisms.

## 4. Conclusions

We demonstrated a bacterial cytoplasmic membrane (BCM) model using vesicles secreted from the Gram-positive bacterial plasma membrane. The suitability of this Gram-positive membrane model to different types of surfaces, including glass, quartz, and PEDOT:PSS, makes this system especially appealing as an alternative to complex cell-based assays or simple phospholipid-based models. This model retains BCM components and recapitulates known drug interactions with the BCM. Our QCM-D and EIS results captured the action of daptomycin on the membrane and demonstrated the changes in viscoelastic properties and permeabilities that result from those interactions. To the best of our knowledge, this is the first report of a Gram-positive bacterial membrane-derived supported lipid bilayer platform for enhanced biosensing applications as a label-free detection tool for antimicrobial activity. Thus, we have established our model system as a platform for elucidating the changes to membrane properties when acted upon by antibiotics. These changes are critical to inform the design and development of emerging antimicrobial compounds that disrupt membranes as a target of action. Given the depleting supply of antibiotics effective in combating bacterial infections, membrane-targeting and membrane-permeabilizing agents have gained traction as tools to overcome the threats of antibiotic resistance. An understanding of the impact of these compounds on the membrane and, in particular, how membrane integrity and permeability are affected, could be studied in-depth using the bilayer platform presented here combined with appropriate bioanalytical tools. Furthermore, the ability to isolate and create bilayers using MVs from clinically relevant Gram-positive isolates would allow us to expand this system to study differences in membrane properties leading to antibiotic susceptibilities and resistance. In the future, combining our SLB system with microfluidic devices can result in the development of high-throughput, label-free biomembrane-based screens for a variety of membrane-targeting molecules. The applications of this membrane model extend beyond antibiotic testing to areas such as host–pathogen interactions, in which the role of membrane components can be studied under specific conditions using our platform, for example, the interactions of phages with bacterial membranes.

## Figures and Tables

**Table 1:** Summary of viscoelastic properties of SLBs.

SLB Composition	Lipid Bilayer (Layer 1)			LTA Layer (Layer 2)		
	Thickness (nm)	Viscosity (cp)	Shear Modulus (kPa)	Thickness (nm)	Viscosity (cp)	Shear Modulus (kPa)
POPC–PEG	6.8 ± 0.9	3.4 ± 0.4	291 ± 43	-	-	-
*B. subtilis*	7.0 ± 0.5	4.4 ± 0.4	876 ± 62	13.7 ± 8.7	1.3 ± 0.0	83 ± 11

## Data Availability

The raw data required to reproduce these findings are available from the corresponding authors upon reasonable request.

## Supplementary Materials

The following supporting information can be downloaded at: https://www.mdpi.com/article/10.3390/bios14010045/s1, S1: Characterization of Gram-positive membrane vesicles (MVs); S2: Mobility of Gram-positive bilayer on different substrates; S3: Estimation of MV rupture percentage; S4: TIRFM images for anti-LTA binding; S5: Modeling QCM-D Data; S6: Fluorescence microscopy imaging of Gram-positive SLBs after daptomycin addition; S7: Electrical characterization of Gram-positive SLB; S8: Changes in Δf and ΔD after daptomycin interaction with Gram-positive bilayer in the presence of Ca^2+^; S9: Daptomycin interaction with Gram-positive bilayer in the absence of Ca^2+^; S10: Daptomycin interaction with POPC–PEG bilayer on QCM-D. Supplementary Video File SV1: Time-lapse video showing *B. subtilis* membrane vesicles (MVs) rupturing on glass upon adding POPC-PEG. MVs labeled with R-18 were adsorbed on the surface and excess vesicles were washed away with buffer before the POPC-PEG liposomes were added to trigger the rupture at the start of the video. When the rupture process starts, the dye molecules start diffusing out of the MVs into the plane of the self-assembled bilayer. Images were taken for a total of 30 min at 1 image/5 s for the first 1 min, then at 1 image/10 s for the next 10 min, and finally at 1 image/min for the remaining period.
