# Supplementary material for: Gram-Positive Bacterial Membrane-Based Biosensor for Multimodal Investigation of Membrane–Antibiotic Interactions
Source: Biosensors (Basel). 2024 Jan 15;14(1):45. doi: 10.3390/bios14010045 (PMC10813107; doi:10.3390/bios14010045)
Supplement: Supplementary file 1 [file biosensors-14-00045-s001.zip › biosensors-2808339-Supplementary.pdf]

Supplementary Information for Gram-Positive Bacterial Membrane-Based Biosensor for Multimodal Investigation of Membrane–Antibiotic Interactions

Samavi Farnush Bint-E-Naser [1], Zeinab Jushkun Mohamed [2], Zhongmou Chao [1], Karan Bali [3], Róisín M. Owens [3] and Susan Daniel [1,*]

[1] Robert F. Smith School of Chemical and Biomolecular Engineering, Cornell University, Ithaca, NY 14853, USA; sb2535@cornell.edu (S.F.B.-E.-N.); zc83@cornell.edu (Z.C.)
[2] Meinig School of Biomedical Engineering, Cornell University, Ithaca, NY 14853, USA; zjm24@cornell.edu
[3] Department of Chemical Engineering and Biotechnology, University of Cambridge, Cambridge CB3 0AS, UK; karankbali@gmail.com (K.B.); rmo37@cam.ac.uk (R.M.O.)
* Correspondence: sd386@cornell.edu

CONTENTS:

S1 Characterization of Gram-positive membrane vesicles (MVs)

S2 Mobility of Gram-positive bilayers on different substrates

S3 Estimation of MV rupture percentage

S4 TIRFM images for anti-LTA antibody binding

S5 Modeling QCM-D Data

S6 Fluorescence microscopy imaging of Gram-positive SLBs after daptomycin addition

S7 Electrical characterization of Gram-positive SLB

S8 Changes in Δf and ΔD after daptomycin interaction with Gram-positive bilayer in the presence of Ca^{2+}

S9 Daptomycin interaction with Gram-positive bilayer in the absence of Ca^{2+}

S10 Daptomycin interaction with POPC-PEG bilayer on QCM-D

S1 Characterization of Gram-positive membrane vesicles (MVs)

Transmission electron microscopy (TEM) and Nanoparticle tracking (NTA, Malvern Nanosight) were used to confirm the size distribution of the isolated vesicles and the results are included in **Figure S1 (a-b)**.

The presence of proteins in the isolated MVs was confirmed using SDS PAGE. ~10^{10} vesicles were mixed with SDS loading dye and heat-treated at 95°C for 5 mins and cooled. Vesicles were then loaded onto 4 - 12% gradient gel using MES buffer. Proteins were stained using Bio-Safe™ Coomassie stain (Bio-Rad Laboratories) and the image of the gel after staining is included in **Figure S1 (c)**.

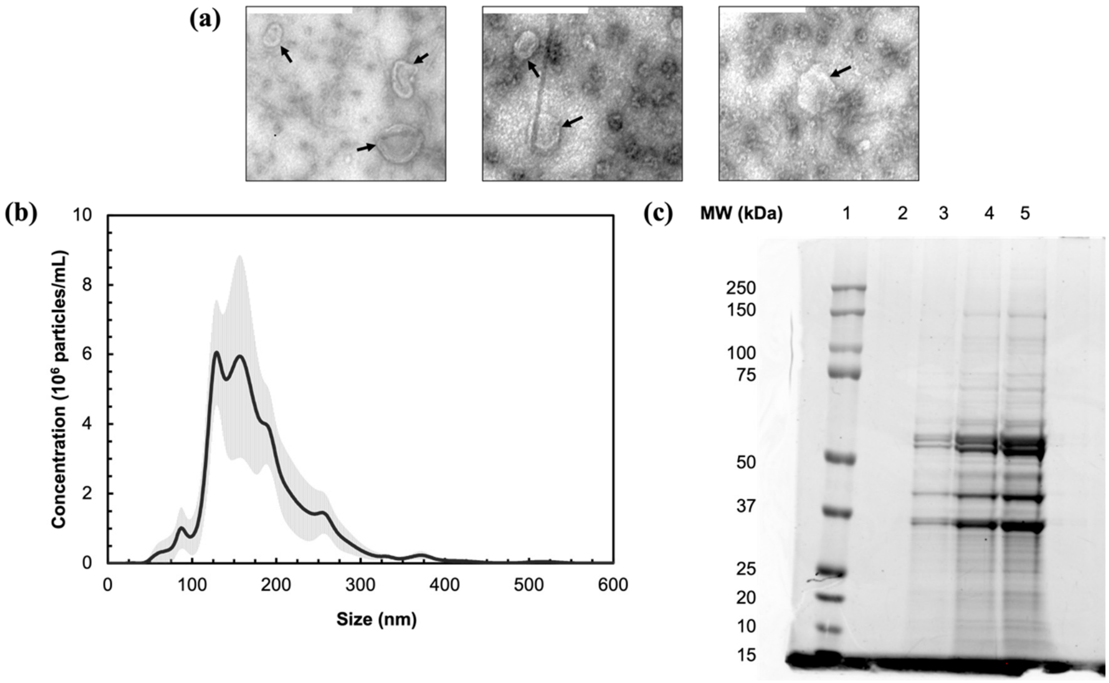

Figure S1. Gram-positive membrane vesicle characterization. (a) Sample TEM images of negatively stained MVs isolated from *B. subtilis* show intact vesicles. Scale bars represent 500 nm. **(b)** Size distribution with respect to particle concentration collected using Nano-particle Tracking Analysis (NTA). Data shows the size of vesicles isolated from *B. subtilis* cells ranged from 119 – 254 nm with an average diameter of 176.8 ± 62.4 nm. This is consistent with TEM and DLS results. Error bars represent standard error. **(c)** SDS-PAGE gel loaded with (lane 1) Ladder, (lane 2) buffer, (lane 3) 3×10^{10} vesicles, (lane 4) 18×10^{10} vesicles, and (lane 5) 3×10^{11} vesicles were stained using Coomassie blue staining kit (Bio-Rad) to visually confirm presence of protein in the MVs.

S2 Mobility of Gram-positive bilayers on different substrates

Surface preparation for optical characterization of Gram-positive SLBs. Microscope coverslips were prepared as described in the **Materials and Methods** section of the main text. Before coating, glass slides were cleaned with oxygen plasma (Harrick Plasma, Ithaca, NY) under

18 W power and 750 m Torr pressure. The PEDOT:PSS solution was formulated with 95 vol % PEDOT:PSS (Clevios PH1000, Heraeus). 5 vol% ethylene glycol (EG), 0.002 vol% dodecylbenzenesulfonic acid (DBSA), and 1 vol% (3-glycidyloxypropyl)trimethyloxy-silane (GOPS). EG, DBSA, and GOPS were obtained from Sigma-Aldrich. The solution was sonicated in an ultrasonic cleaner (VWR) and passed through a 0.45 μm filter before spin coating at 2500 rpm for 35 s. Following coating, the slides were annealed at 140 °C for 1 h and hydrated for 4 h in DI water before drying under a stream of ultra-high purity nitrogen and stored at room temperature for use. Prior to use, PEDOT:PSS coated slides were cleaned with O_2 plasma for 1 min using the above-mentioned conditions.

Optical verification of Gram-positive SLB formation. To aid in visualizing bilayers, Gram-positive MVs were labeled with Octadecyl Rhodamine B (R18), a membrane intercalating dye. 0.5 - 0.7 μL of fluorophore solution containing 0.5 mg/mL R18 in ethanol was added to 400 μL of particle solution and placed in a bath sonicator (VWR) for 15 mins at 20°C. Excess dye was removed from the suspension using Microspin G-25 columns (GE) at 150 g for 3 mins. Fluorescently labeled vesicles were then used to form bilayers following the protocol described in the **Materials and Methods** section of the main text on both glass and PEDOT: PSS-coated glass coverslips. For the Gram-positive bilayer, the rupture of adsorbed vesicles after POPC-PEG addition was recorded to confirm the formation of a continuous, planar bilayer on the different supports.

Figure S2 illustrates the vesicle rupture process for bilayer formation using POPC–PEG as rupture vesicles on both glass and PEDOT:PSS. When unruptured MVs are adsorbed on the surface, the dye confined within them remains quenched **(Figure S2a**). Upon the addition of unlabeled POPC–PEG liposomes, the vesicles start rupturing and R18 is no longer quenched within the membrane of the vesicles (**Figure S2b**). As the dye becomes free to diffuse through the lipids in the surrounding area, the overall fluorescence of the sample increases and becomes uniform with time as the bilayer formation process is completed (**Figure S2c**). The ability to successfully form planar, supported bilayers using the bacterial MVs on both non-conducting and conducting surfaces expands the utility of this platform to carry out electrical characterization in addition to fluorescent assays to study membrane interactions.

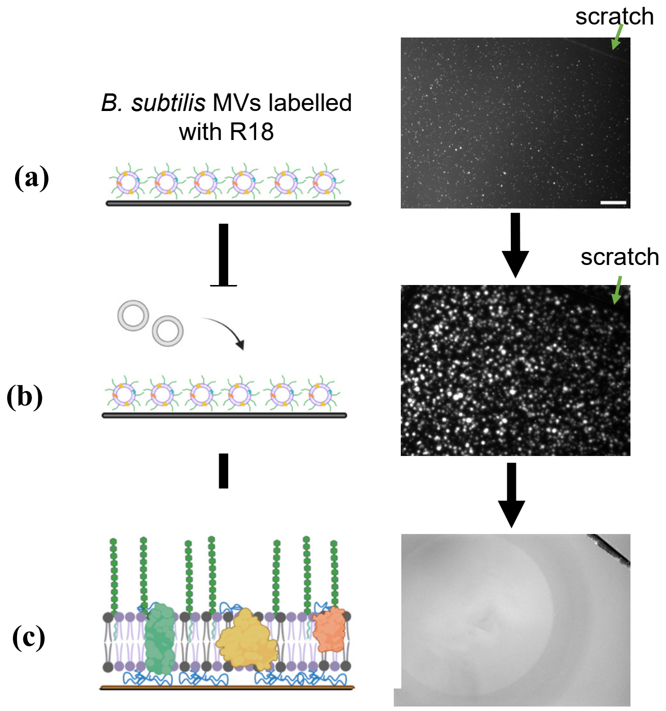

Figure S2. Formation of Gram-positive SLB. (a) R18 contained in adsorbed vesicles show up as punctate spots when imaged. (b) As POPC-PEG is added, it induces rupture of the adsorbed vesicles and R18 starts diffusing out from the vesicles across the plane of the bilayer as it forms. (c) The completion of the rupture process is indicated by the homogeneous distribution of the R18 fluorescence throughout the bilayer. Vesicles and their components in the figures on the left are not drawn to scale. For images on the right, scale bar represents 50 µm.

Diffusion kinetics determination. Fluorescence recovery after photobleaching (FRAP) was used to confirm mobility and determine diffusion kinetics of SLBs formed on glass and PEDOT:PSS surfaces. The two-dimensional diffusivity was measured by continuously monitoring a photobleached area on the bilayers as the fluorescence recovered. An inverted Zeiss Axio Observer.Z1 microscope with α Plan-Apochromat 20× objective was used for imaging. A ~ 20 μm diameter spot on the focal plane of the SLBs was photobleached using a 150 mW 561 nm laser. The recovery of the photobleached spot was recorded for 30 mins. With time, the fluorescence of the bleached spot recovered for the Gram-positive bilayers on both glass and PEDOT:PSS surfaces (**Figure S3**). By fitting the recorded data with a Bessel function following the method reported by Soumpasis *et al.*[1] for the recovery of a two-dimensional circle, we were able to extract diffusion coefficient (D) and mobile fraction (M.F.) values for our samples (**Figure S3**). D was calculated using the equation:

$$D = \frac{w^2}{t_{1/2}} \quad \text{(S1)}$$

where w and $t_{1/2}$ represent the radius of the photobleached spot and the time required to achieve half of the maximum recovery intensity, respectively.

The diffusion coefficient of R18 in the Gram-positive bilayers was found to be ~ 0.43 μm^2/s on both glass and PEDOT:PSS. These results along the high M.F. values (95 - 99%) demonstrate the retention of mobility in the Gram-positive SLBs and confirm the formation of a contiguous bilayer on a micron scale.

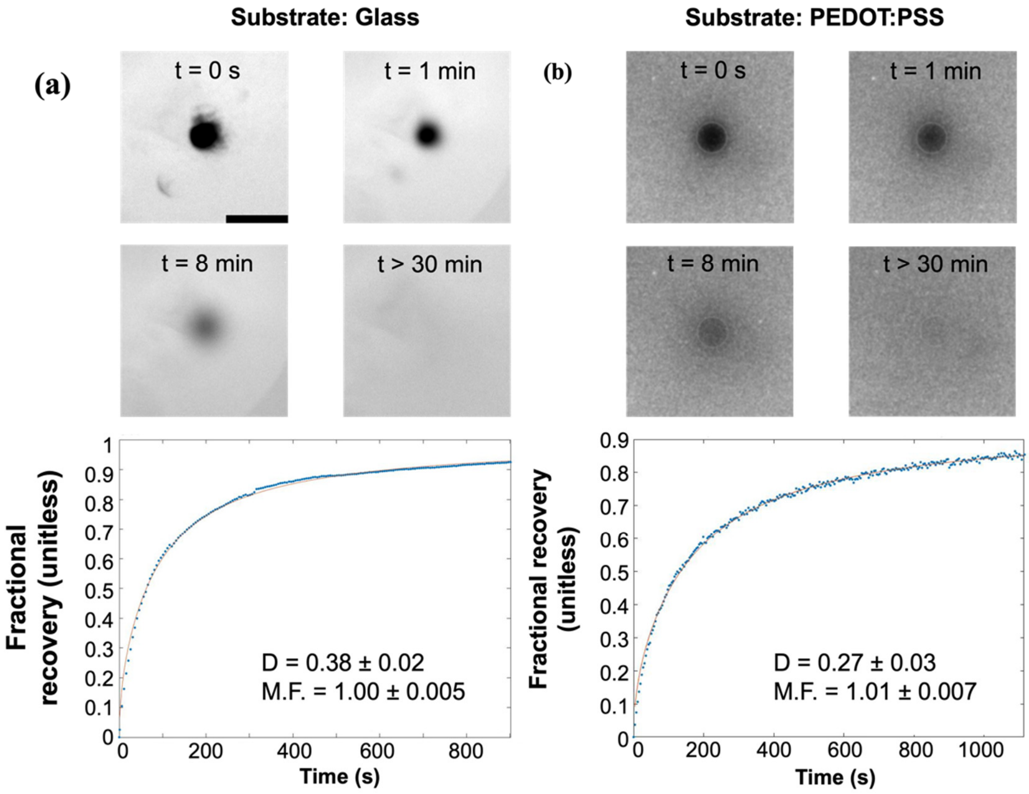

Figure S3. Mobility of Gram-positive bilayer on different substrates. Recovery of fluorescence of a ~20 µm photobleached spots on the focal plane of the bilayers on **(a)** glass and **(b)** PEDOT:PSS with time are shown on top. Bottom plots demonstrate representative fit of fractional recovery of fluorescence over time and the calculated values of 2*D* diffusion coefficient (*D*) of R18 and mobile fraction (M.F.) for all SLBs. Blue circles represent experimental data and red lines represent data fitted to a Bessel function. The scale bar represents 50 µm.

S3 Estimation of MV rupture percentage

The rupture percentage of *B. subtilis* vesicles during SLB formation on QCM-D has been estimated following the method published by Hsia et. al. [2]. For this, first, we assessed the surface coverage (θ) of MVs adsorbed on the sensor surface using the following equation:

$$\theta = \frac{0.54 \times Mass\ of\ adsorbed\ MVs\left(\frac{ng}{cm^2}\right), M_{Ad}}{Saturation\ mass\ of\ adsorbed\ MVs\left(\frac{ng}{cm^2}\right), M_{Ad-sat}} \quad \text{(S2)}$$

To determine the denominator, we formed a saturated monolayer of adsorbed MVs on the sensor and approximated the jamming mass using the one-layer Voigt model which was approximately 4000 ng/cm^2 (**Figure S4**). The mass of adsorbed MVs during bilayer formation was also estimated using the same model to be ~1400 ng/cm^2. The jamming limit of spheres on a 2D plane (0.54) [3] along with the mass of adsorbed MVs were used to determine the surface coverage by the vesicle. Since the coupled water mass may change with the number of vesicles adsorbed on the sensor surface, the θ value determined using eqn. **S2** was corrected using a 3D COMSOL Multiphysics model [2] developed based on the theoretical model by Bingen et. al. [4] (θ = 0.14).

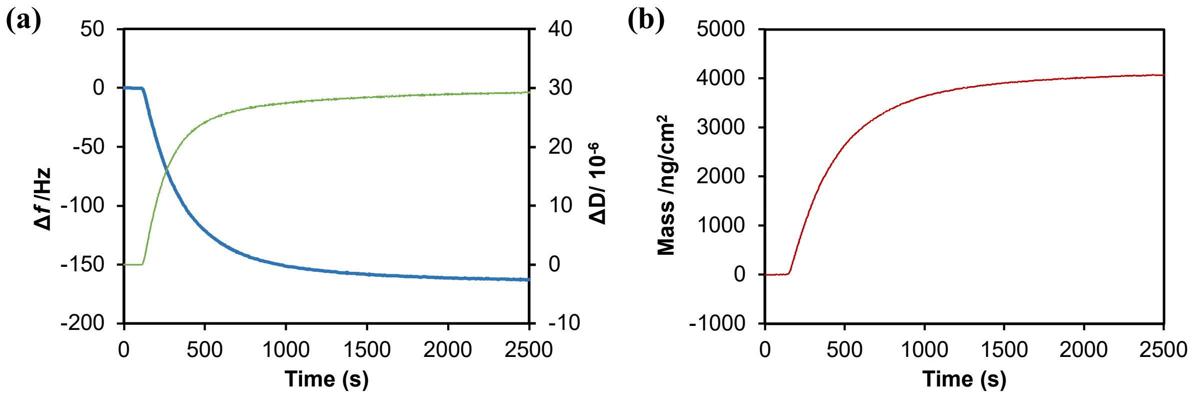

Figure S4. (a) Change in frequency Δf (blue) and dissipation ΔD (green) recorded during MV adsorption to create saturated layer on the QCM-D sensor. (b) The data was used to estimate the saturation mass of MVs using the one-layer Voigt model.

Next, the theoretical mass of a pure MV bilayer was predicted. For this, we considered the contributions of lipids, protein, and LTA polymers. Using the data for the lipid bilayer from **Table 1** and considering 9:1 lipid-to-protein ratio by weight, the mass of the lipid bilayer along with their coupled solvent was estimated to be ~825 ng/cm^2. Finally, the mass contribution from the LTA molecules was estimated to be ~1100 ng/cm^2 considering the molecular weight of 8500 kDa [5] and 12 mol% LTA in the vesicle [6]. Thus, the mass of an SLB made with only vesicles from *B. subtilis* membrane, $M_{MV\text{-}SB}$ was calculated to be 1930 ng/cm^2. This value was used for determining the theoretical mass of a Gram-positive SLB considering 100% rupture of the adsorbed vesicles using the following equations:

$$M_{MV-SB,T} = M_{MV-SB} \times \kappa\theta + M_{POPC-PEG-SLB} \times (1 - \kappa\theta) \quad \text{(S3)}$$

$$\kappa = \frac{Surface\ area\ of\ ruptured\ MVs}{Projected\ area\ of\ unruptutred\ MVs} = \frac{4\pi r^2}{\pi r^2} \quad \text{(S4)}$$

Finally, MV rupture percentage, P was estimated using the experimentally determined mass of the Gram-positive SLB as follows:

$$M_{MV-SB,Exp} = (1 - P) \times M_{ad} + M_{MV-SB} \times \kappa P\theta + M_{POPC-PEG-SLB} \times (1 - (1 - P)\theta - \kappa\theta)$$

(S5)

When $P \rightarrow 1$, $M_{MV-SB,Exp} = M_{MV-SB,T}$

The experimental mass of the Gram-positive SLB estimated from the two-layer model (1560 ng/cm^2) was used in **equation S5** to check and correct the rupture percentage. We determined the rupture percentage to be ~78% and the surface coverage by native material to be ~54%.

S4 TIRFM images for anti-LTA antibody binding

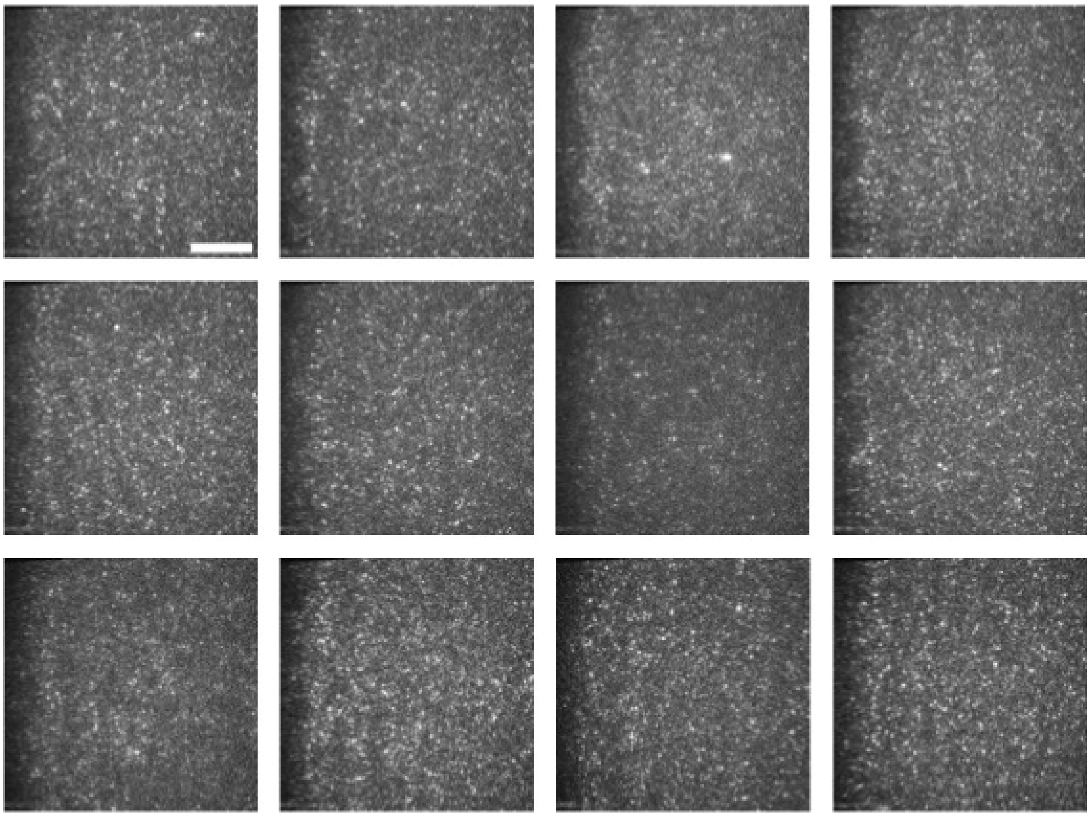

Figure S5. TIRFM images for anti-LTA antibody binding to Gram-positive bilayers show retention of LTA. Images represent both technical and biological replicates. The scale bar represents 20 μm.

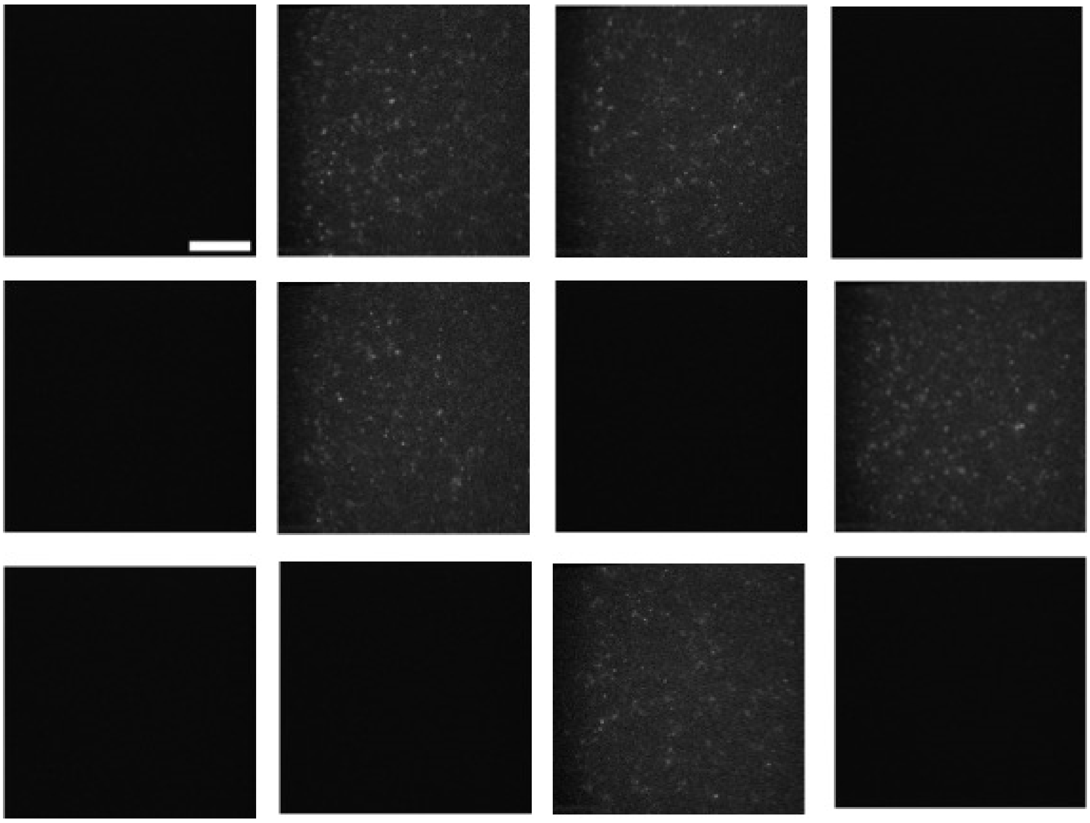

Figure S6. TIRFM images for anti-LTA antibody binding to POPC-PEG bilayers show minimum interference from the lipids used for rupture. Images represent both technical and biological replicates. The scale bar represents 20 μm.

S5 Modeling QCM-D Data

For biological samples on QCM-D sensors, the ΔD values are often greater than 10^{-6} requiring the application of viscoelastic models to estimate properties such as thickness, viscosity, and shear modulus. While the one-layer models (Kelvin-Voigt or Maxwell) can be used to capture the viscoelastic behavior of homogeneous films, the Gram-positive bilayers containing both native membrane materials and POPC-PEG components are inhomogeneous. Therefore, we applied the two-layer Voigt-Voinova model [7] for estimating the viscoelastic properties of the native material-derived bilayers while the one-layer model was used for the POPC-PEG bilayer, based on previously published work [8,9]. This model analyzes layered films on the sensor surface based on Voigt assumption of spring and dashpot in parallel. The Δf and ΔD are calculated by using the following equations:

$$\Delta f \approx \frac{1}{2\pi\rho_0 h_0}\left\{\sum_{j=1,2}\left[\rho_j h_j \omega - 2h\left(\frac{\eta_3}{\delta_3}\right)^2\left(\frac{\eta_j \omega^2}{\mu_j^2 + \eta_j^2\omega^2}\right)\right]\right\} \quad \text{(S6)}$$

$$\Delta h \approx \frac{1}{2\pi f \rho_0 h_0}\left\{\sum_{j=1,2}\left[2h\left(\frac{\eta_3}{\delta_3}\right)^2\left(\frac{\mu_j \omega}{\mu_j^2 + \eta_j^2\omega^2}\right)\right]\right\} \quad \text{(S7)}$$

The following parameters were used for solving equations S6 and S7:

ρ_0 = quartz crystal density

h_0 = quartz sensor thickness = 334 um [10]

$\rho_0 h_0$ = 0.885 kg/m^3

η_3 = shear viscosity of fluid (1*10^{-3} kg/m.s)

δ_3 = bulk fluid penetration depth at fundamental frequency (~250 nm for fluid density of 1000 kg/m^3)

ω = angular frequency = $2\pi f$

ρ_1 = film density of layer 1 (1100 kg/m^3)

ρ_2 = film density of layer 2 (600 kg/m^3)

The film densities were estimated based on the native material content in the Gram-positive bilayers (54%) assessed in section **S3**. Contributions from the POPC-PEG (ρ = 1100 kg/m^3), bacterial lipids (ρ = 1100 kg/m^3), and proteins (ρ = 1400 kg/m^3) were considered for layer 1. For layer 2, densities of LTA (ρ ~ 1800 kg/m^3) [11] and water ((ρ = 1000 kg/m^3) were considered.

The calculated Δf and ΔD were fit to experimental Δf and ΔD values at various overtones (3rd, 5th, 7th, 9th, 11th, and 13th) to solve for shear modulus (μ_1, μ_2), viscosity (η_1, η_2), and thickness (h_1, h_2) of the top and bottom layers. The fitting was performed using MATLAB *fmincon* function (MathWorks) which determined the minimum value of the constrained nonlinear equation:

$$\Delta F\ (\mu_1, \mu_2, \eta_1, \eta_2, h_1, h_2) \approx \sum\nolimits_{n=3,5,7,9,11,13} \left(\Delta f_n^{experimental} - \Delta f_n^{calculated}\right)^2 + \left(\Delta D_n^{experimental} - \Delta D_n^{calculated}\right)^2 \qquad \text{(S8)}$$

To determine the global minima for the system from the local minima obtained by solving the nonlinear function F, viscoelastic parameters were constrained to appropriate physical values:

$$8.9 \times 10^{-4}\ \text{Pa.s} \leq \eta \leq 1\times10^{-1}\ \text{Pa.s}$$

$$10^4\ \text{Pa} \leq \mu \leq 5\times10^6\ \text{Pa}$$

$$10^{-9}\ \text{m} \leq h \leq 10^{-7}\ \text{m}$$

Solutions contained within those constraints and with the lowest difference between experimental and calculated Δf and ΔD were considered for viscoelastic solutions for the system. We estimated the properties of the Gram-positive SLB before and after interaction with daptomycin in the presence of Ca^{2+} by analyzing and fitting specific changes in Δf and ΔD to the model equations above.

To demonstrate the appropriateness of the two-layer model for predicting the viscoelastic behavior of the Gram-positive SLB, we also used the one-layer to fit the Δf and ΔD for this system. **Table S1** contains a comparison of the properties of this SLB estimated with both models and **Figure S7** includes the fit for the changes in frequency and dissipation of the Gram-positive bilayer to both models and the POPC-PEG SLB to the one-layer model. These show that the two-layer model provides a better fit to the Δf and ΔD data collected using QCM-D and the properties estimated by this model are biologically significant.

Table S1. Estimation of viscoelastic properties of Gram-positive SLB.

**SLB composition**	**Lipid bilayer (Layer 1)**			**LTA layer (Layer 2)**		
	**Thickness (nm)**	**Viscosity (cp)**	**Shear modulus (kPa)**	**Thickness (nm)**	**Viscosity (cp)**	**Shear modulus (kPa)**
B. subtilis	13.7 ± 1.5	1.8 ± 0.1	162 ± 37	-	-	-
	7.0 ± 0.5	4.4 ± 0.4	876 ± 62	16.3 ± 8.7	1.3 ± 0.0	83 ± 11

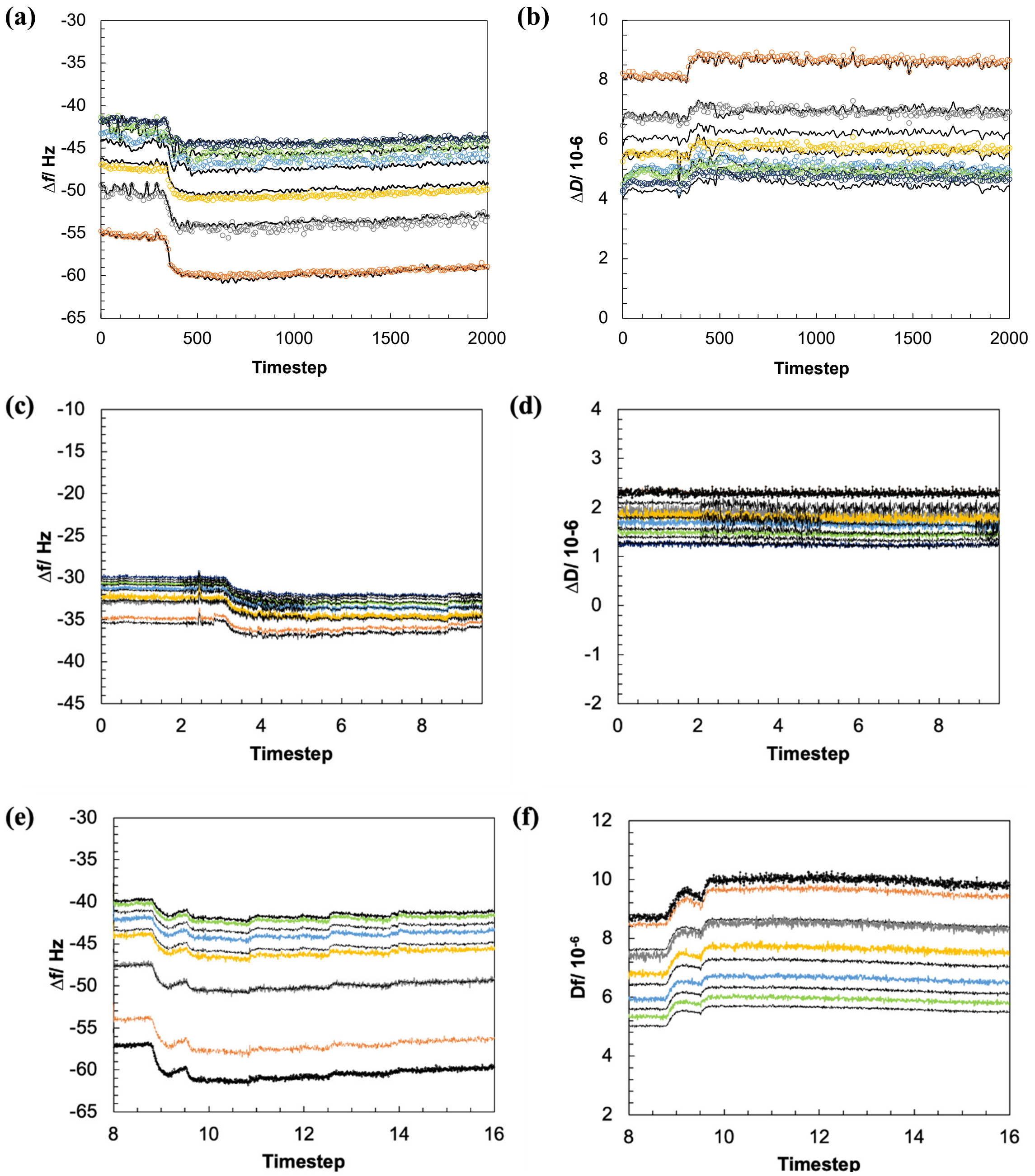

Figure S7. Representative dataset showing model fit to QCM-D results for addition of Daptomycin in presence of Ca^{2+}. (a-b) Fit to the two-layer model for (a) Δf and (b) ΔD values for the Gram-positive bilayer. (c-d) Fit to the one-layer model for (c) Δf and (d) ΔD values for the POPC-PEG bilayer. (e-f) Fit to the one-layer model for (e) Δf and (f) ΔD values for the Gram-positive bilayer. Different colors in different overtones: orange (3rd = 15 MHz), grey (5th = 25 MHz), yellow (7th = 35 MHz), light blue (9th = 45 MHz), green (11th = 55 MHz), and dark blue (13th = 65 MHz). Calculated Δf and ΔD using the one-layer or the two-layer models are shown in black solid lines. The calculated values align well with experimental data. Data shown in these plots are not normalized.

S6 Fluorescence microscopy imaging of Gram-positive SLBs after daptomycin addition

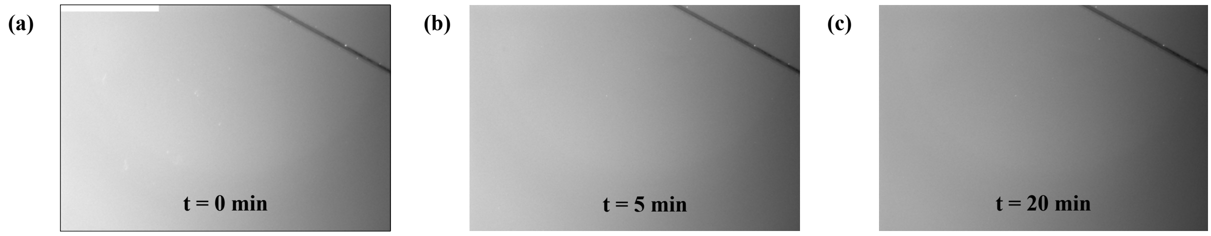

Figure S8. No phase separation was observed upon Daptomycin addition to Gram-positive SLBs at the scale of diffraction limit (200-250 nm) . (a) R-18 labelled vesicles were used to form Gram-positive SLBs on glass (t = 0 min). (b-c) Images were taken using an inverted Zeiss Axio Observer.Z1 microscope with α Plan-Apochromat 20× objective at regular intervals after introducing daptomycin supplemented with Ca^{2+}. No lipid de-mixing was observed even after 20 mins. The scale bar represents 100 µm.

S7 Electrical characterization of Gram-positive SLB

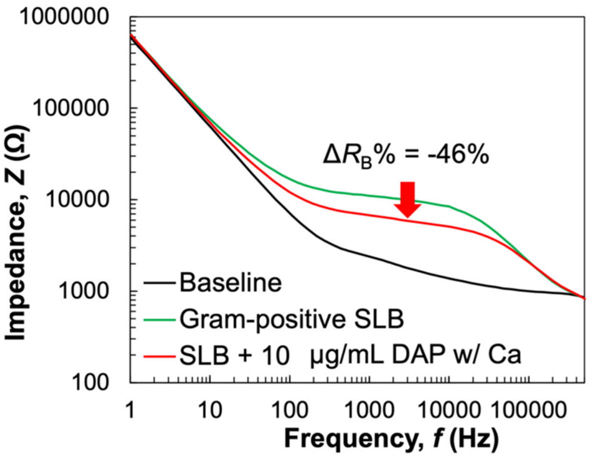

Figure S9. EIS monitoring of SLB interactions with daptomycin. Representative Bode plot showing impedance response of Gram-positive SLB upon addition of 10 µg/mL daptomycin (DAP) in the presence of Ca^{2+}

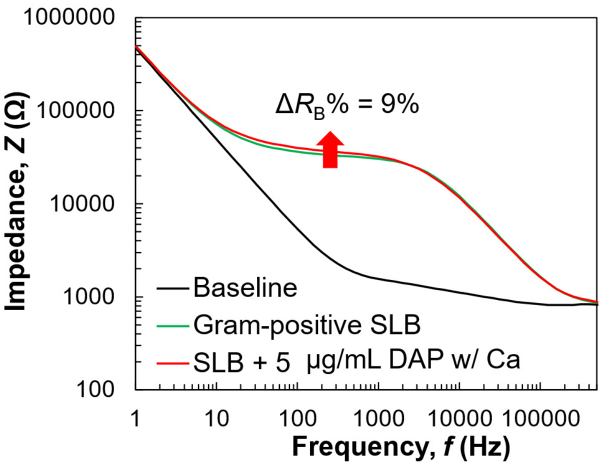

Figure S10. Representative Bode plot for EIS monitoring of SLB interactions with daptomycin. SLB formed using Gram-positive vesicles and POPC lipids in 1:50 ratio electrode of 420 µm. High POPC fractions in the SLB results in a decreased membrane response due to the lack of interaction between POPC and (5 µg/mL) daptomycin (DAP) in the presence of Ca^{2+}.

Table S2. Electrical properties of SLBs and changes in membrane resistance after interactions with daptomycin.

SLB composition	Condition	Resistance, R_B	Capacitance, C_B	ΔR_B%*
POPC	5 µg/mL Daptomycin with Ca^{2+}	41.8 ± 11.6	1.1 ± 0.2	9.4 ± 3.2
Gram-positive	1 µg/mL Daptomycin with Ca^{2+}	6.8 ± 3.9	1.1 ± 0.4	-15.1 ± 7.8
	5 µg/mL Daptomycin with Ca^{2+}			-40.7 ± 10.9
	10 µg/mL Daptomycin with Ca^{2+}			-40.1 ± 15.4
	5 µg/mL Daptomycin without Ca^{2+}			10.6 ± 4.2

$$* \Delta R_B = \frac{R_{B,DAP} - R_{B,SLB}}{R_{B,SLB}}$$

where $R_{B,SLB}$ = SLB resistance before antibiotic addition
and $R_{B,DAP}$ = SLB resistance after interactions with the antibiotic

S8 Changes in Δf and ΔD after daptomycin interaction with Gram-positive bilayer in the presence of Ca^{2+}

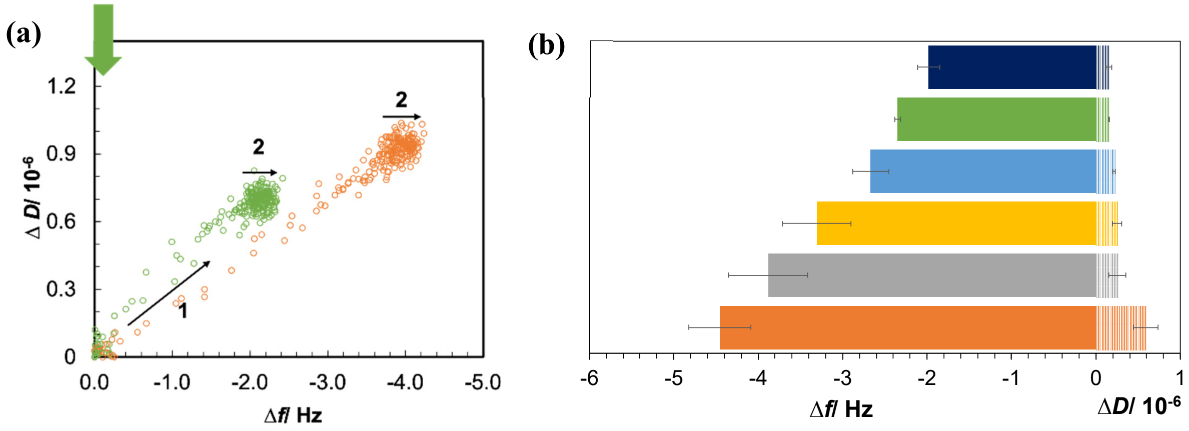

Figure S11. Representative plots monitoring the interaction of daptomycin with Gram-positive SLB in the presence of Ca^{2+}. (a) Plots of Δf vs. ΔD for the 3rd (orange) and 11th (green) overtones show Daptomycin-membrane interaction is a two- step process. The initial changes (marked as 1 in the figure) show antibiotic adsorption (drop in Δf) results in rapid loss of membrane rigidity (rise in ΔD) with subsequent changes (marked as 2 in the figure) showing daptomycin insertion and aggregation into the membrane (further drop in Δf). Changes are more pronounced at the lower overtone (shown in orange) indicating the antibiotic affects the upper leaflet more. (b) Changes in Δf (solid bars to the left) and ΔD (patterned bars to the right) values of SLB after addition of the antibiotic indicate that the vertical distribution of daptomycin across the bilayer is non-homogeneous distribution of daptomycin as daptomycin-Ca^{2+} complexes interact with accessible PG lipids on the outer leaflet of the membrane and the lipopeptide antibiotic inserts itself partially into the lipid bilayer. For both plots, different colors represent different overtones: orange (3rd = 15 MHz), grey (5th = 25 MHz), yellow (7th = 35 MHz), light blue (9th = 45 MHz), green (11th = 55 MHz), and dark blue (13th = 65 MHz). Values are normalized with respect to initial values at each overtone.

S9 Daptomycin interaction with Gram-positive bilayer in the absence of Ca^{2+}

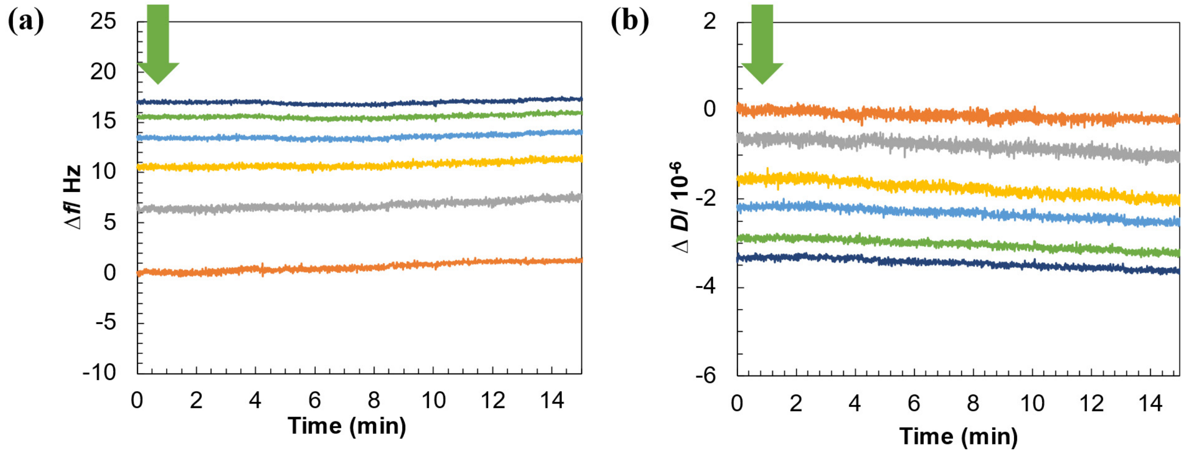

Figure S12. Specificity of antibiotic interaction with *B. subtilis* SLB. The SLB washed with buffer without Ca^{2+} before daptomycin addition. Representative plots monitoring daptomycin interaction with SLB in the absence of Ca^{2+}. No significant changes in (a) Δf and (b) ΔD values were observed upon antibiotic addition (marked with green arrows). Different colors in a-c represent different overtones: orange (3rd = 15 MHz), grey (5th = 25 MHz), yellow (7th = 35 MHz), light blue (9th = 45 MHz), green (11th = 55 MHz), and dark blue (13th = 65 MHz).

S10 Daptomycin interaction with POPC-PEG bilayer on QCM-D

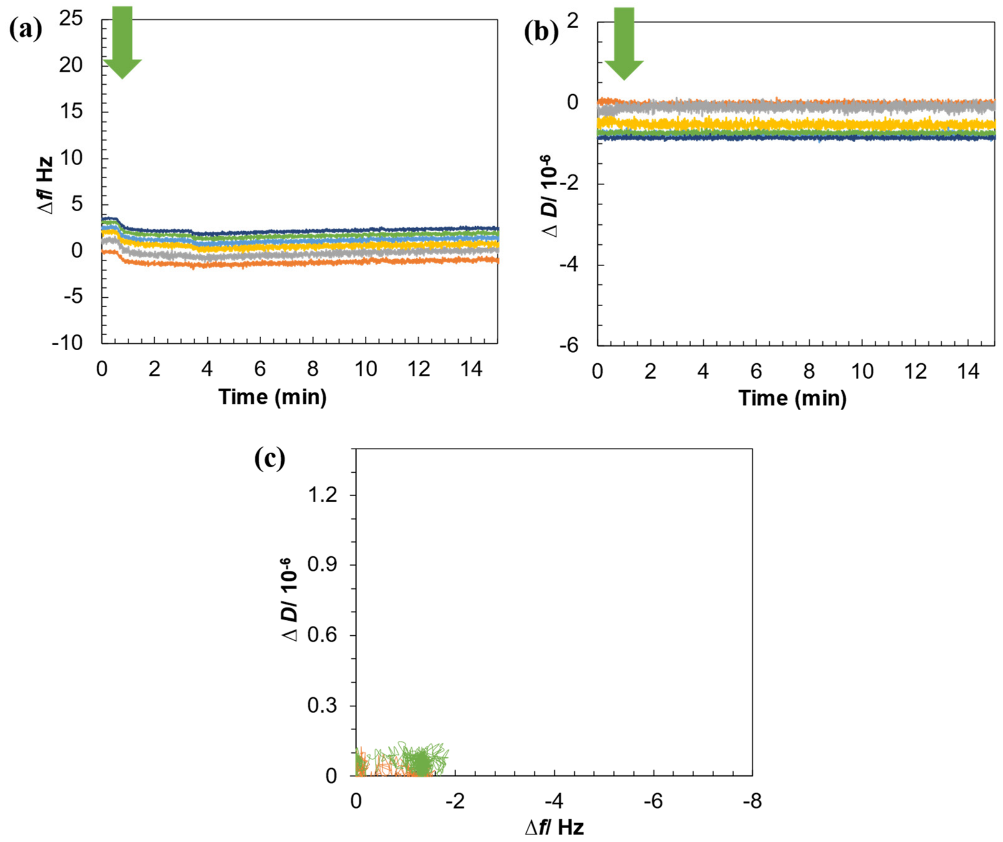

Figure S13. Representative plots monitoring the interaction of daptomycin with POPC-PEG. The SLB washed with buffer supplemented with Ca^{2+} before daptomycin addition. Minimal changes were observed in (a) Δf and (B) ΔD for all overtones upon addition of the antibiotic in the presence of Ca^{2+} (green arrows mark the start of Daptomycin flow). (c) Δf vs. ΔD trend after Daptomycin addition to POPC-PEG bilayers show no loss in bilayer viscoelasticity or rigidity indicating no antibiotic mediated disruption occur in this case. Different colors represent different overtones: orange (3rd = 15 MHz), grey (5th = 25 MHz), yellow (7th = 35 MHz), light blue (9th = 45 MHz), green (11th = 55 MHz), and dark blue (13th = 65 MHz).

References

1. Soumpasis, D.M. Theoretical Analysis of Fluorescence Photobleaching Recovery Experiments. *Biophys. J.* **1983**, *41*, 95–97, doi:10.1016/S0006-3495(83)84410-5.

2. Hsia, C.-Y.Y.; Chen, L.; Singh, R.R.; DeLisa, M.P.; Daniel, S. A Molecularly Complete Planar Bacterial Outer Membrane Platform. *Sci. Rep.* **2016**, *6*, 1–14,

doi:10.1038/srep32715.

3. Cho, N.-J.; Wang, G.; Edvardsson, M.; Glenn, J.S.; Hook, F.; Frank, C.W. Alpha-Helical Peptide-Induced Vesicle Rupture Revealing New Insight into the Vesicle Fusion Process As Monitored in Situ by Quartz Crystal Microbalance-Dissipation and Reflectometry. *Anal. Chem.* **2009**, *81*, 4752–4761, doi:10.1021/ac900242s.
4. Bingen, P.; Wang, G.; Steinmetz, N.F.; Rodahl, M.; Richter, R.P. Solvation Effects in the Quartz Crystal Microbalance with Dissipation Monitoring Response to Biomolecular Adsorption. A Phenomenological Approach. *Anal. Chem.* **2008**, *80*, 8880–8890, doi:10.1021/ac8011686.
5. Fischer, W.; Rösel, P. The Alanine Ester Substitution of Lipoteichoic Acid (LTA) in Staphylococcus Aureus. *FEBS Lett.* **1980**, *119*, 224–226, doi:10.1016/0014-5793(80)80257-2.
6. Bharatiya, B.; Wang, G.; Rogers, S.E.; Pedersen, J.S.; Mann, S.; Briscoe, W.H. Mixed Liposomes Containing Gram-Positive Bacteria Lipids: Lipoteichoic Acid (LTA) Induced Structural Changes. *Colloids Surfaces B Biointerfaces* **2021**, *199*, 111551, doi:10.1016/j.colsurfb.2020.111551.
7. Voinova, M. V; Rodahl, M.; Jonson, M.; Kasemo, B. Viscoelastic Acoustic Response of Layered Polymer Films at Fluid-Solid Interfaces: Continuum Mechanics Approach. *Phys. Scr.* **1999**, *59*, 391–396, doi:10.1238/Physica.Regular.059a00391.
8. Hsia, C.Y.; Chen, L.; Singh, R.R.; DeLisa, M.P.; Daniel, S. A Molecularly Complete Planar Bacterial Outer Membrane Platform. *Sci. Rep.* **2016**, *6*, doi:10.1038/srep32715.
9. Mohamed, Z.; Shin, J.-H.; Ghosh, S.; K. Sharma, A.; Pinnock, F.; Bint E Naser Farnush, S.; Dörr, T.; Daniel, S. Clinically Relevant Bacterial Outer Membrane Models for Antibiotic

Screening Applications. *ACS Infect. Dis.* **2021**, *7*, 2707–2722, doi:10.1021/acsinfecdis.1c00217.

10. Edvardsson, M. The QCM Fundamental Frequency – How It Matters in Measurements Available online: https://www.biolinscientific.com/blog/does-the-qcm-fundamental-frequency-matter.

11. ChemSpider D-Ribitol 5-Phosphate Available online: https://www.chemspider.com/Chemical-Structure.133184.html (accessed on 1 January 2024).
